# Heat Stress Modulates WDR5‐Mediated H3K4me3 Modification to Induce Melanogenesis via Activating CX3CL1/CX3CR1 Axis

**DOI:** 10.1002/advs.202510164

**Published:** 2025-11-20

**Authors:** Yushan Zhang, Ling Jiang, Yibo Hu, Chuhan Fu, Jinhua Huang, Jing Chen, Qinghai Zeng

**Affiliations:** ^1^ Department of Dermatology The Third Xiangya Hospital Central South University Changsha 410013 China; ^2^ Clinical Research Center The Second Xiangya Hospital Central South University Changsha 410011 China

**Keywords:** CX3CL1, CX3CR1, H3K4me3, heat stress, melanogenesis, MYC, WDR5

## Abstract

The skin's unique thermosensitivity renders it exceptionally responsive to thermal perturbations, wherein heat stress exposure disrupts cutaneous homeostasis and activates pigmentary pathways. While clinical observations consistently link heat stress to hyperpigmentation disorders, the precise molecular mechanism for heat‐induced melanogenesis‐particularly the epigenetic‐immune crosstalk mediating this process‐constitutes an unmet research challenge. Transcriptomic analysis of melasma reveals a significant positive correlation between CX3CL1 and melanogenesis. Consistently, CX3CL1 significantly enhances melanogenesis in both cultured skin tissues and melanocytes. Mechanistically, CX3CL1 increases melanogenesis through CX3CR1‐dependent activation of JNK signaling pathway. Notably, heat stress triggers recruitment of WDR5, a core subunit of the H3K4me3 methyltransferase, to the CX3CL1 promoter. At this site, WDR5 catalyzes H3K4me3 modification, thereby driving the transcription of CX3CL1. Inhibition of WDR5 expression can reverse the heat stress‐induced upregulation of H3K4me3 enrichment level in the CX3CL1 promoter region, thereby suppressing CX3CL1 expression and subsequently reducing melanogenesis. Further investigation reveals that the recruitment of WDR5 to the CX3CL1 promoter is MYC‐dependent. In conclusions, heat stress promotes melanogenesis by upregulating CX3CL1 through the MYC‐WDR5‐H3K4me3 axis, thereby activating the CX3CL1/CX3CR1‐JNK signaling pathway. This study elucidates the mechanism by which heat stress regulates skin pigmentation and reveals novel targets with therapeutic potential for pigmentary skin disorders.

## Graphical abstract

This study elucidates the mechanism by which heat stress regulates skin pigmentation: heat stress upregulates CX3CL1 through the MYC‐WDR5‐H3K4me3 axis, thereby activating the CX3CL1/CX3CR1‐JNK signaling pathway and ultimately promoting melanogenesis. These findings provide novel potential therapeutic targets for pigmentary skin disorders.

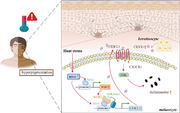

## 1 Introduction

Pigmentary dermatoses, such as vitiligo and melasma, are among the most prevalent and disfiguring diseases in dermatology.^[^ ^1^, ^2^ ^]^ These diseases predominantly affect young and middle‐aged individuals, and their disfiguring nature often leads to significant psychological distress and social impairment.^[^ ^3^ ^]^ The resulting high demand for treatment and patients' elevated expectations imposes considerable pressure on healthcare systems, a challenge that cannot be overlooked. The pathogenesis of these disorders is closely linked to dysregulated melanogenesis in melanocytes.^[^ ^4^, ^5^ ^]^ Environmental factors, including ultraviolet radiation, temperature fluctuations, and PM2.5 exposure, are well‐established stimulators of melanogenesis.^[^ ^6^, ^7^, ^8^ ^]^ Notably, as extreme heat events become more frequent and intense, the role of temperature in exacerbating pigmentary disorders‐and its broader implications for human health‐has drawn increasing attention.

Skin is highly sensitive to temperature changes, long‐term or repeated local heat stress can lead to the occurrence of erythema ab igne, and local heat stress is an important cause of melasma.^[^ ^9^, ^10^ ^]^ Our previous study shown that heat promotes melanogenesis by increasing the paracrine effects in keratinocytes via the TRPV3/Ca2+/Hh pathway.^[^ ^8^ ^]^ Emerging evidence has established the inflammatory microenvironment as a critical modulator of melanogenesis.^[^ ^11^ ^]^ Inflammatory factors, particularly IL‐1, IL‐7, IL‐15, IFN‐γ and TNF‐α demonstrate bidirectional regulatory capacity in melanogenesis.^[^ ^12^, ^13^, ^14^, ^15^, ^16^ ^]^ In contrast, the role of chemokines in regulating melanogenesis remains poorly understood. Notably, the potential immuno‐pigmentary crosstalk underlying heat stress‐mediated pigmentation remains underexplored, particularly regarding how heat stress may transcriptionally regulate melanogenic‐inflammatory factors through epigenetic events.

CX3CL1(C‐X3‐C motif chemokine ligand 1) is the only member of the CX3C chemokine family, which only via its unique receptor CX3CR1, is involved in the occurrence and development of cancer,^[^ ^17^ ^]^ kidney disease,^[^ ^18^ ^]^ and nervous system diseases.^[^ ^19^ ^]^ In addition, CX3CL1 is also involved in disease progression in the skin, the CX3CL1‐CX3CR1 axis plays a key role in the initiation of skin cancer by regulating the aggregation and function of macrophages through IL‐1 and TNF‐α.^[^ ^20^, ^21^ ^]^ However, the involvement of CX3CL1 in skin pigmentation remains unexplored. Intriguingly, accumulating evidence indicates that CX3CL1 expression is highly susceptible to external stimuli,^[^ ^22^, ^23^, ^24^ ^]^ suggesting that CX3CL1 may as a potential key mediator in heat stress‐induced skin hyperpigmentation.

This study systematically investigates the regulatory role of CX3CL1 in melanogenic processes and its response to heat stress. Through comprehensive mechanistic analyses, we elucidate the involvement of CX3CL1 in melanogenesis signaling pathways while concurrently characterizing its epigenetic modulation under heat stress exposure. Our study reveals novel molecular cross‐talk between heat stress and pigmentary regulation, establishing CX3CL1 as a key mediator in cutaneous pigment homeostasis.

## 2 Results

### 2.1 CX3CL1 Expression was Positively Correlated with the Melanin Content in Human Skin

**Figure 1:**
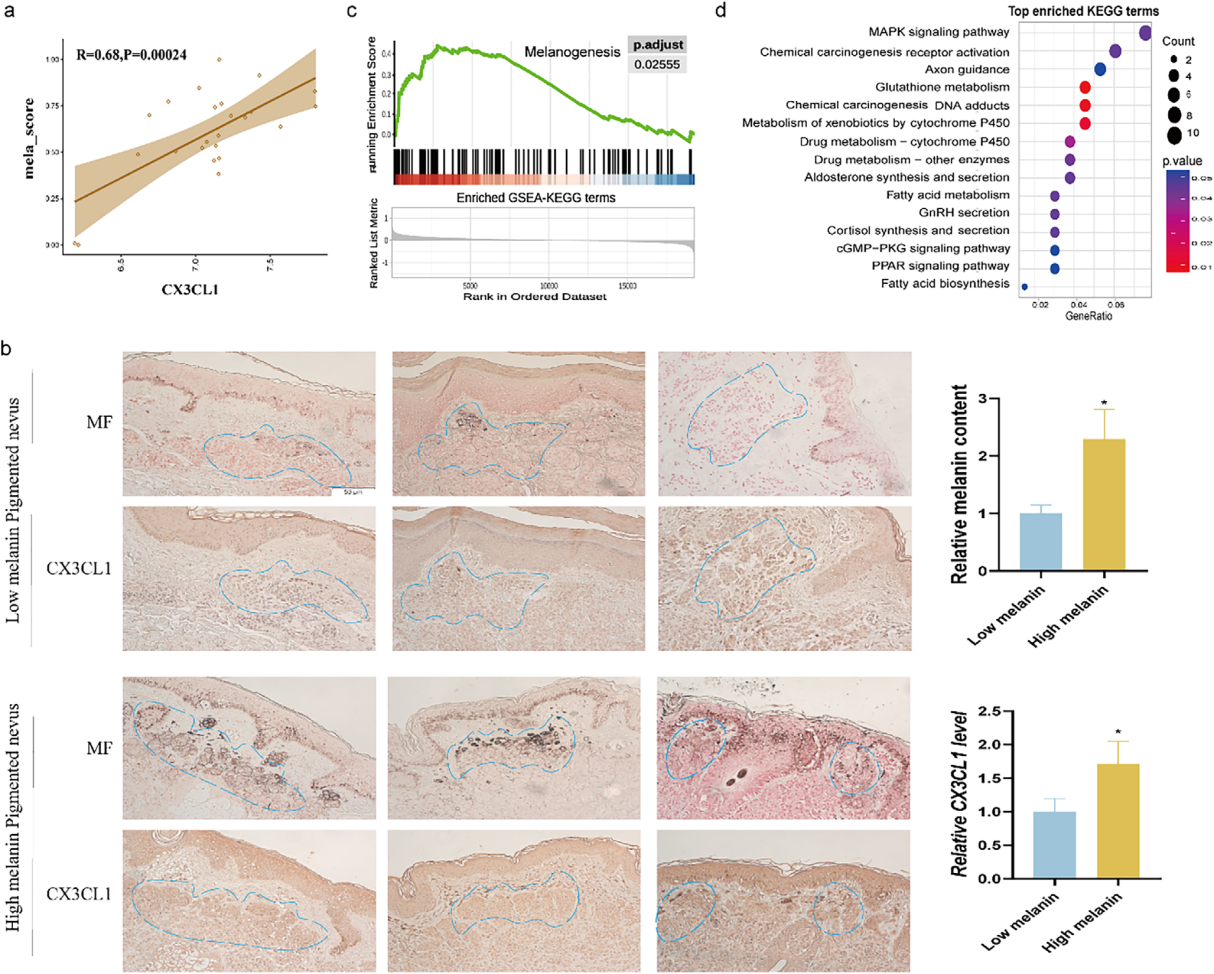
CX3CL1 expression was positively correlated with the melanin content in human skin. a) The correlation between CX3CL1 and mela‐score in GSE72140. b) Representative images of tissue sections stained with Fontana‐Masson (MF) and IHC showing melanin granules and CX3CL1 protein expression in the skin tissue, and the melanocyte nests are circled by the blue dotted lines (Scale=50µm). Quantitative analysis was conducted using ImageJ. c,d) The results of GSEA (c) and KEGG (d) enrichment analysis in GSE72140. Statistical analysis was conducted via Unpaired Student's t‐test, mean ± SEM, n = 3 (^**^ *p* < 0.05).

To investigate whether CX3CL1 plays a regulatory role in skin pigmentation, we analyzed the RNA expression data of melasma (GSE72140)^[^ ^28^ ^]^ skin samples. We defined key melanogenesis‐related genes (MITF, TYR, TYRP1, and DCT) as a functional gene set of melanogenesis, and the weighted score (mela‐score) of this gene set was calculated by single‐sample gene set enrichment analysis (ssGSEA). We found that there was a strong positive correlation between CX3CL1 expression and the mela‐score in dataset (**Figure** **1**a). Consistent with this, we analyzed skin samples of low‐melanin content pigmented nevus and high‐melanin content pigmented nevus, immunohistochemistry revealed that CX3CL1 was relatively highly expressed in high‐melanin content pigmented nevus (Figure 1b). To gain further insights into the functional role of CX3CL1, the melasma samples in GSE72140 were divided into CX3CL1^high^ and CX3CL1^low^ groups based on the median expression of CX3CL1. The differentially expressed genes (DEGs) between the two groups were screened (Figure S1, Supporting Information), and gene set enrichment analysis (GSEA) showed that melanogenesis gene set was enriched in the CX3CL1^high^ group (Figure 1c). Meanwhile, the top 200 DEGs (ordered by P‐value) were subjected to Kyoto Encyclopedia of Genes and Genomes (KEGG) analysis, which showed significant enrichment of the MAPK signaling pathway (Figure 1d). The information of all datasets is provided in Table S3 (Supporting Information). The detailed bioinformatics analysis methods were conducted as previously studies described.^[^ ^11^ ^]^

### 2.2 CX3CL1 can Promote Melanogenesis

**Figure 2:**
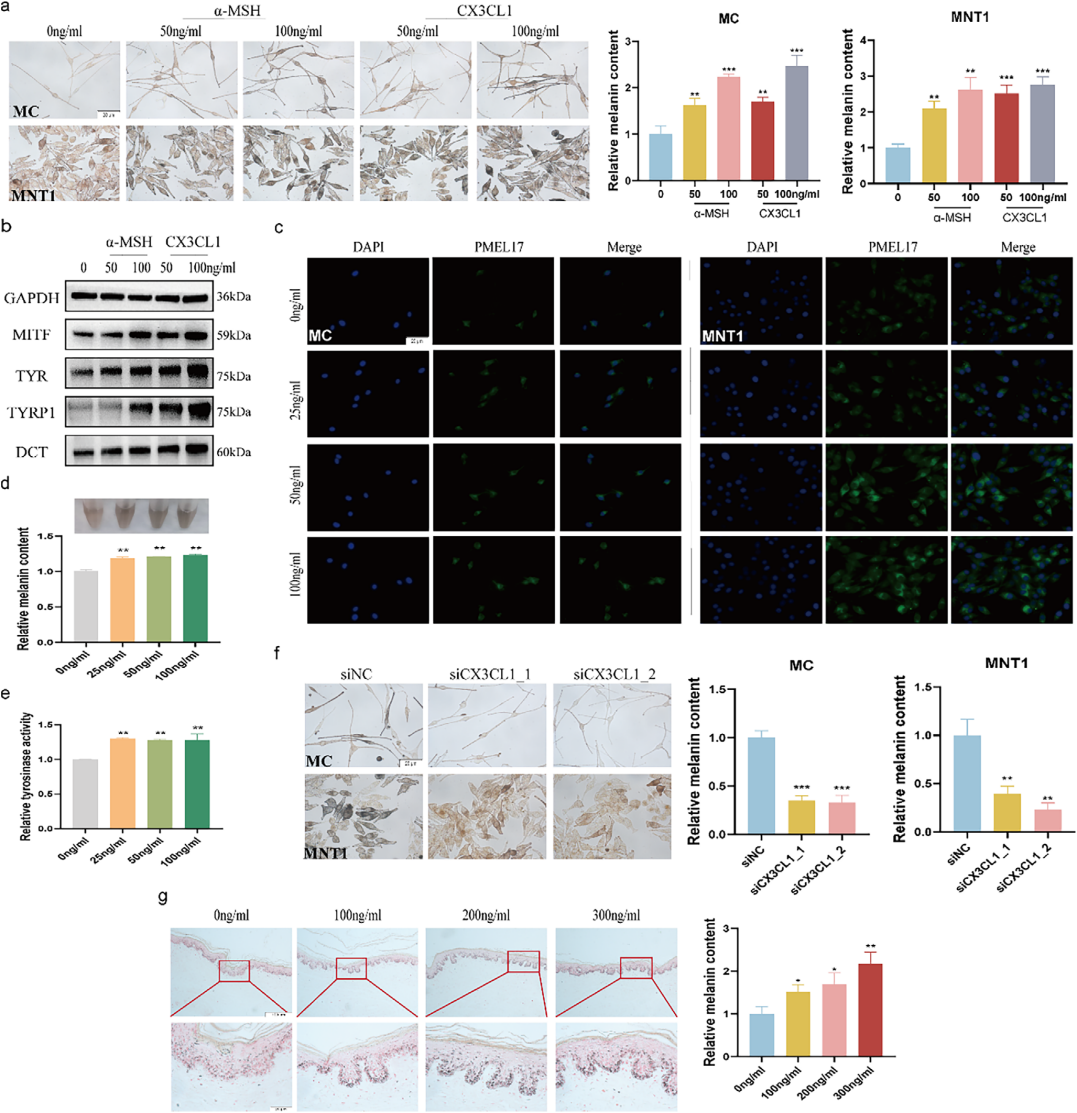
CX3CL1 can promote melanogenesis. a,b) MNT1 or MC cells were treated with α‐MSH or CX3CL1: after 24 h, the melanin content in the cells was detected by Fontana‐Masson staining (scale =20 µm), and quantitative analysis was performed using ImageJ (a); after 48 h, the protein expression of key melanogenesis‐related genes in MNT1 cells was detected by Western blot(b). c–e) After MNT1 or MC cells treated with CX3CL1 for 48h: representative images showing in situ expression of PMEL17(c) (Scale=20µm); Melanin content in the MNT1 cells was measured by the NaOH method (d); Tyrosinase activity in the MNT1 cells was measured by L‐DOPA assay (e). f) si_CX3CL1 was transfected into MC and MNT1 cells for 24 h, the intracellular melanin content was detected by Fontana‐Masson staining (Scale = 20 µm). g) Representative images of Fontana‐Masson staining of human foreskin tissue stimulated with CX3CL1 for 5 days (Scale = 20 µm and 100 µm), and quantitative analysis was conducted using ImageJ. Statistical analysis was conducted via a one‐way ANOVA with multiple comparisons, mean ± SEM, n = 3 (^*^ *p* < 0.05, ^**^ *p* < 0.01, and ^***^ *p* < 0.001).

Bioinformatics analysis indicates that CX3CL1 may positively regulate melanogenesis. Subsequently, we observed the effect of CX3CL1 on human primary cells (MC) and melanin‐rich human melanoma cell line MNT1. CX3CL1 did not affect the viability of the cells at concentrations below 300ng mL^−1^ (Figure S2a, Supporting Information). Meanwhile, different concentrations of CX3CL1 significantly increased the melanin content in the melanocytes (Figure S2b, Supporting Information). Notably, CX3CL1 proved to be as potent as α‐MSH,^[^ ^29^ ^]^ a widely recognized stimulator of melanogenesis, at equal concentrations (Figure 2a). Similar to α‐MSH, CX3CL1 significantly upregulated the protein expression of key melanogenesis‐related genes, including MITF, TYR, TYRP1, and DCT (Figure 2b; Figure S2c, Supporting Information). After MC or MNT1 was treated with CX3CL1, the expression of the melanosome marker PMEL17 was significantly upregulated (Figure 2c). Consistent with the upregulation of melanogenic genes, we observed a significant increase in both intracellular melanin content and tyrosinase activity (Figure 2d,e), as quantified by the NaOH and L‐DOPA methods, respectively. Silencing of CX3CL1 (Figure S2d–f, Supporting Information) significantly reduced the intracellular melanin content (Figure 2f), confirming its positive regulatory role in melanogenesis.

The aforementioned results demonstrate that CX3CL1 can directly enhance melanogenesis in melanocytes. Previous studies have shown that inflammatory factors can indirectly regulate melanogenesis by modulating fibroblasts or keratinocytes through paracrine signaling.^[^ ^12^, ^30^ ^]^ Accordingly, we further investigated whether CX3CL1 regulates melanogenesis through a paracrine mechanism. When MNT1 cells were co‐cultured with human immortalized keratinocytes (HaCaT) or human dermal fibroblasts (FB), the exogenous addition of CX3CL1 still resulted in an increase in melanin content within MNT1 cells (Figure S3a,b, Supporting Information). Similarly, we also observed that the conditioned medium from CX3CL1‐treated keratinocytes or fibroblasts significantly enhanced the melanin content in MNT1 cells (Figure S3c–f, Supporting Information). In addition, the human foreskin tissues stimulated with exogenous CX3CL1 also showed an increase in melanin content (Figure 2g). It is noteworthy that CX3CL1 has the capacity to modulate the expression of various inflammatory cytokines, such as TNF‐α, IL‐6, and IL‐18,^[^ ^31^, ^32^ ^]^ which have been previously implicated in the regulation of melanogenesis.^[^ ^14^, ^15^, ^33^ ^]^ To determine whether the effect of CX3CL1 on melanogenesis extends beyond its direct action, we profiled the expression of multiple related inflammatory cytokines that are regulated by CX3CL1 and implicated in pigmentation. Our findings revealed that following exogenous addition of CX3CL1 to skin tissue, the expression levels of IL‐4, IL‐6, IL‐18, and IL‐37 were significantly upregulated, while TNF‐α expression remained unchanged (Figure S4, Supporting Information). The above results indicate that CX3CL1 may regulate melanogenesis by modulating other inflammatory factors, implicating a broader cytokine network that requires further clarification.

### 2.3 CX3CL1 Regulates Melanogenesis via the Receptor CX3CR1

**Figure 3:**
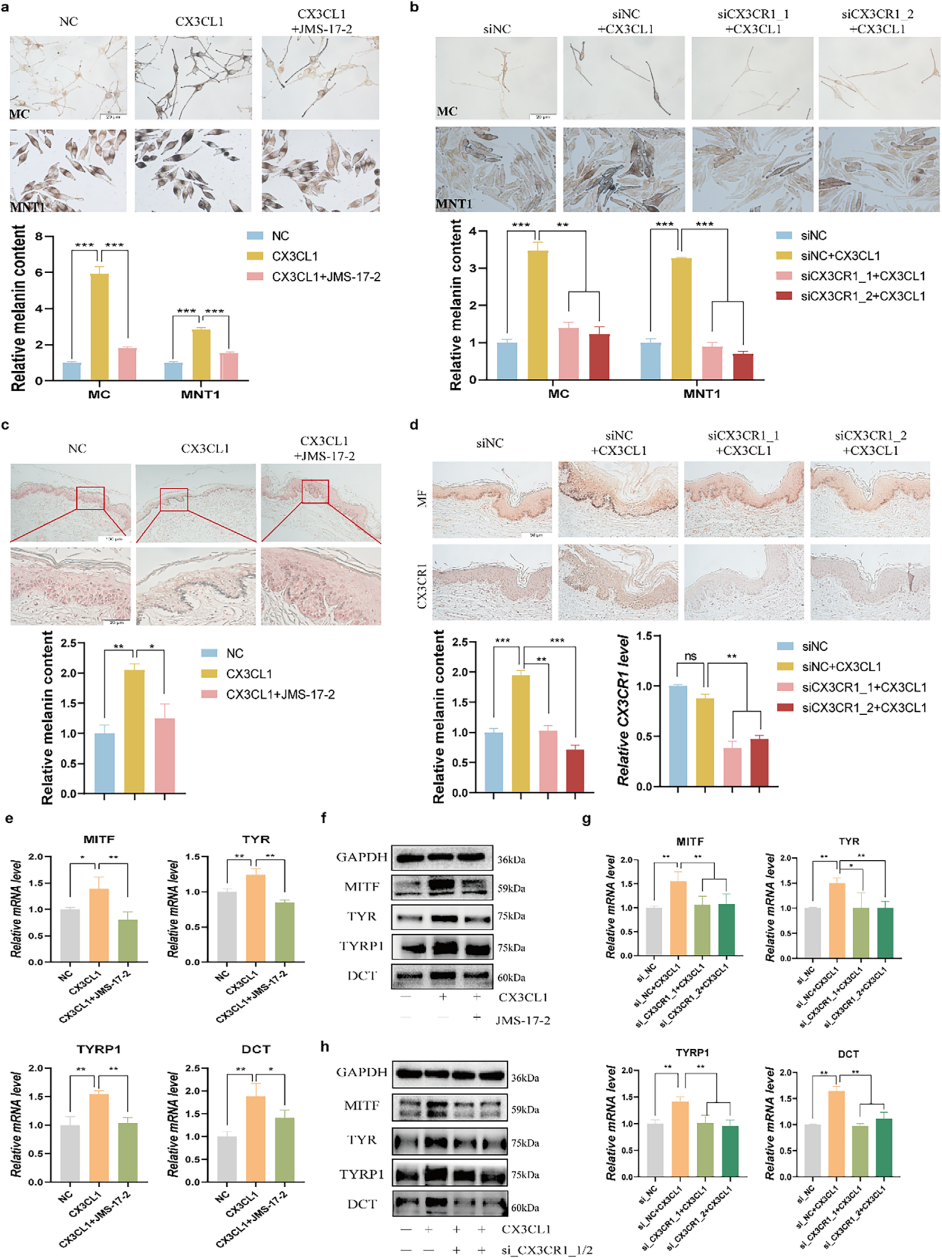
CX3CL1 regulates melanogenesis via the receptor CX3CR1. a,b) After the MC and MNT1 cells pretreated with JMS‐17‐2(10nm) for 30min (a) or transfected with siCX3CR1 (b) were then treated with CX3CL1(100 ng mL^−1^) for 24h, the changes in melanin content in the cells were detected by Fontana‐Masson staining (Scale=20µm). Quantitative analysis was conducted using ImageJ. c,d) After the skin tissues pretreated with JMS‐17‐2 for 30min (c) or with low expression of CX3CR1(d) were co‐treated with CX3CL1 for 5 days, the changes in melanin content in the skin tissues were detected by Fontana‐Masson staining, and quantitative analysis was conducted using ImageJ. e,f) The MNT1 cells pretreated with JMS‐17‐2 for 30 min were further co‐treated with CX3CL1, the expression of melanogenesis key genes was analyzed by qRT‐PCR after 24h (e), and by Western blot after 48h (f). g,h) After treating CX3CR1‐low‐expressing MNT1 cells with CX3CL1, the expression of melanogenesis key genes was assessed by qRT‐PCR at 24h (g), and by Western blot at 48h (h). Statistical analysis was conducted via a one‐way ANOVA with multiple comparisons, mean ± SEM, n = 3 (ns: no significant difference, ^*^ *p* < 0.05, ^**^ *p* < 0.01, and ^***^ *p* < 0.001).

CX3CR1 is the sole receptor for CX3CL1. To further verify whether CX3CL1 promotes melanogenesis via the receptor CX3CR1, human foreskin tissue and melanocytes were pretreated with non‐toxic concentrations of the CX3CR1 inhibitor JMS‐17‐2 (10 nm) or siCX3CR1 (Figure S5a,b, Supporting Information), followed by stimulation with CX3CL1 (100 ng mL**^−1^**). The results demonstrated that both the application of the CX3CR1‐specific antagonist JMS‐17‐2 and the suppression of CX3CR1 expression via siCX3CR1 (Figure S5c–e, Supporting Information) could effectively neutralize the CX3CL1‐induced increase in melanin content in melanocytes and human skin tissues (**Figure** **3**a–d). Notably, JMS‐17‐2 alone similarly reduced melanin content in melanocytes in a concentration‐dependent manner (Figure S5f, Supporting Information). Furthermore, suppression of CX3CR1 abolished the CX3CL1‐mediated increase in melanin content across multiple cell lines, including human immortalized melanocytes (PIG1) and melanin‐rich melanoma cells (MEWO, A2058) (Figure S5g,h, Supporting Information). Subsequently, both JMS‐17‐2 and silencing CX3CR1 expression can antagonize the CX3CL1‐induced upregulation of key melanogenesis‐related genes in MNT1 cells (Figure 3e–h).

### 2.4 CX3CL1‐CX3CR1 Affects Melanogenesis by Regulating the JNK Signaling Pathway

**Figure 4:**
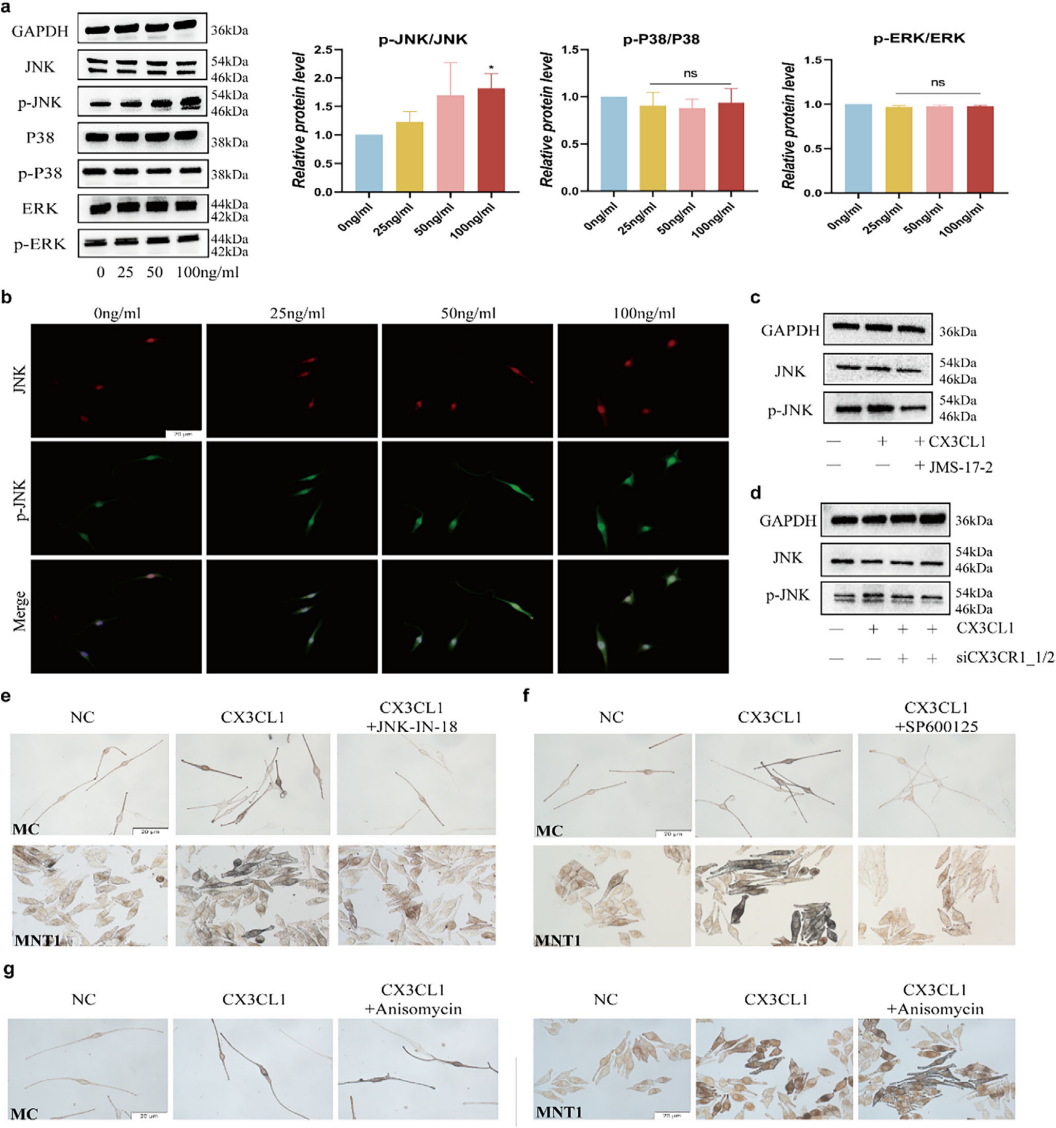
CX3CL1‐CX3CR1 affects melanogenesis by regulating the JNK signaling pathways. a,b) After treating MNT1 or MC cells with CX3CL1 for 4h: Western blot showing expression of key proteins of MAPK pathways in MNT1 cells (a); IF shows the protein expression of JNK and p‐JNK in MC cells (b). c,d) MNT1 cells pretreated with JMS‐17‐2 for 30min(c) or transfected with siCX3CR1 for 48h (d), and then treated with CX3CL1 for 4h, the protein expressions of JNK and p‐JNK were detected by Western blot. e,f) After MC and MNT1 cells pretreated with 1 µm JNK‐IN‐18 (e) or 5 µM SP600125 (f) for 30 min, and then treated with CX3CL1 for 24h. The changes in melanin content were detected by Fontana‐Masson staining. g) After MC and MNT1 cells pretreated with 10nm anisomycin for 30min, and then treated with CX3CL1 for 24h. The changes in melanin content were detected by Fontana‐Masson staining. Scale = 20 µm. Statistical analysis was conducted via a one‐way ANOVA with multiple comparisons, mean ± SEM, n = 3 (ns: no significant difference, ^*^ *p* < 0.05).

MAPK is a key signaling pathway that promotes melanogenesis. Furthermore, the CX3CL1‐CX3CR1 axis is involved in various inflammatory processes through its interaction with the MAPK pathways.^[^ ^34^ ^]^ Consistent with this, the MAPK signaling pathways were enriched in the CX3CL1^high^ group in the GSE72140 dataset (Figure 1d). Therefore, we further explored the changes in the key proteins of these pathways in the CX3CL1‐treated cells. Time‐course analysis of CX3CL1‐stimulated MNT1 cells showed that JNK phosphorylation was significantly increased, beginning at 4 h post‐treatment (Figure S6, Supporting Information). However, a 4‐h treatment with various concentrations of CX3CL1 did not alter the phosphorylation levels of ERK and p38 (**Figure** **4**a). In the CX3CL1‐treated MC cells, we also observed the high level of p‐JNK (Figure 4b). JMS‐17‐2 and silencing CX3CR1 expression attenuated the effects of CX3CL1 on the phosphorylation level of JNK (Figure 4c,d). Furthermore, we used JNK inhibitors (JNK‐IN‐18 and SP600125) to inhibit the JNK signaling pathway for functional restoration. JNK‐IN‐18 and SP600125 can reverse CX3CL1‐induced increase in melanin content (Figure 4e,f). Meanwhile, activation of the pathway using the JNK agonist anisomycin^[^ ^35^ ^]^ revealed a synergistic effect with CX3CL1, resulting in a further increase in melanin content in cells (Figure 4g).

### 2.5 Heat Stress can Stimulate the Expression of CX3CL1

**Figure 5:**
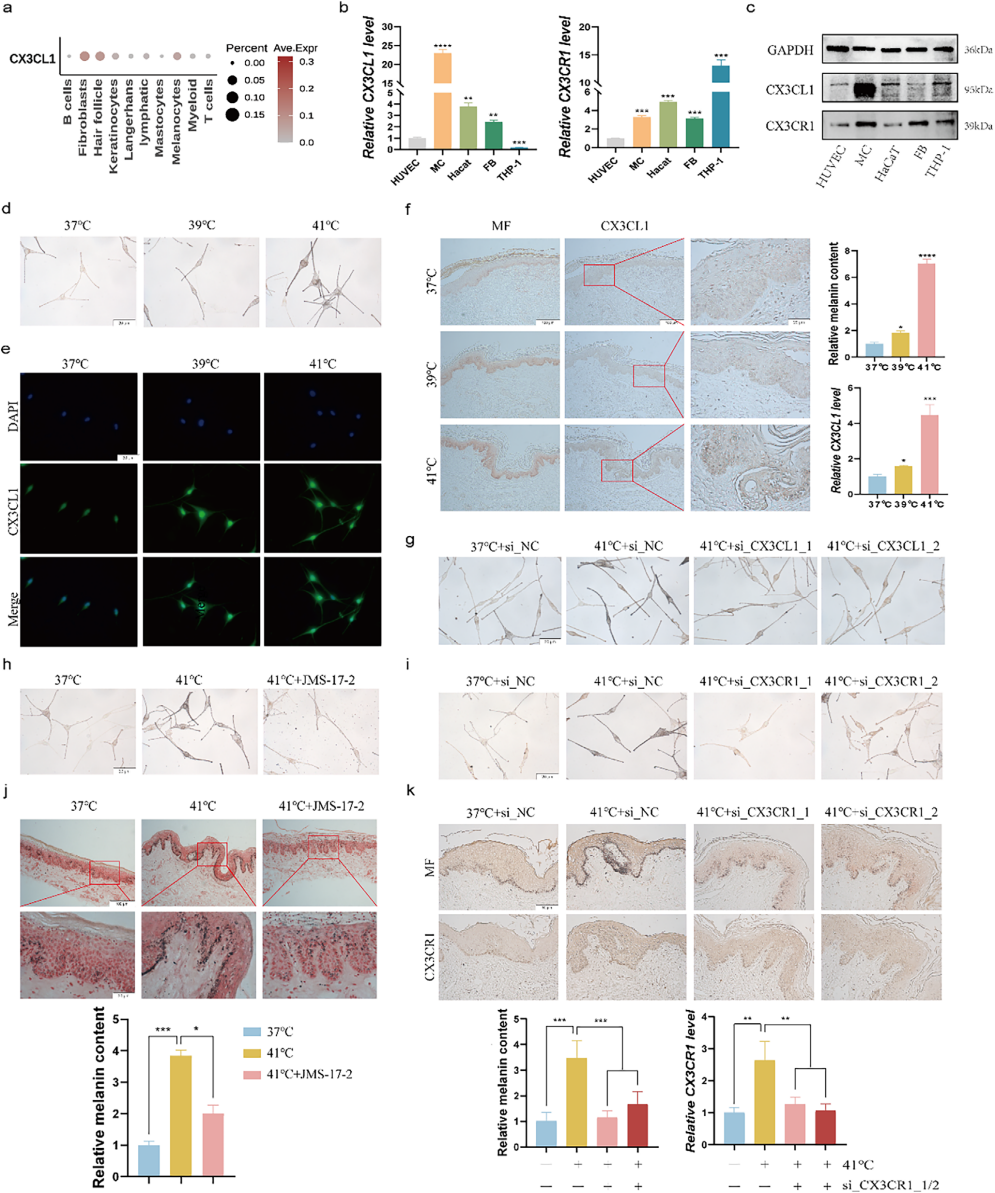
Heat stress can stimulate the expression of CX3CL1. a)The expression of CX3CL1 in each cell in the single‐cell data GSE150672. b,c) The mRNA and protein levels of CX3CL1 and CX3CR1 in multiple cells were detected by qRT‐PCR (b) and Western blotting (c). d,e) After MC cells were exposed to heat stress for 3 days: Fontana‐Masson staining was used to detect the melanin content (d); IF was used to detect the expression of CX3CL1 (e) (Scale = 20 µm). f) Representative images of tissue sections stained with Fontana‐Masson and IHC showing melanin granules and CX3CL1 protein expression in the skin tissue exposed to heat stress for 5 days (Scale = 20 µm and 100 µm), and quantitative analysis was conducted using ImageJ. g) MC cells transfected with siCX3CL1 were exposed to heat stress for 3 days, the changes in melanin content were detected by Fontana‐Masson staining (Scale = 20 µm). h,i) After the MC cells pretreated with JMS‐17‐2 for 30min(h) or transfected with siCX3CR1(i) were exposed to heat stress for 3 days, the changes in melanin content were detected by Fontana‐Masson staining (Scale = 20 µm). j,k) After the skin tissues pretreated with JMS‐17‐2 for 30 min (j) or with low expression of CX3CR1(k) were exposed to heat stress for 5 days, the changes in melanin content in the skin tissues were detected by Fontana‐Masson staining, and quantitative analysis was conducted using ImageJ. Statistical analysis was conducted via a one‐way ANOVA with multiple comparisons, mean ± SEM, n = 3 (^*^ *p* < 0.05, ^**^ *p* < 0.01, and ^***^ *p* < 0.001).

The human epidermal melanocyte unit (EMU) is essential for skin pigmentation.^[^ ^36^ ^]^ Therefore, we analyzed the expression of CX3CL1 in melanocytes and keratinocytes by analyzing the single‐cell dataset GSE150672.^[^ ^37^ ^]^ As shown in **Figure** **5**a, CX3CL1 was expressed at relatively high levels in both melanocytes and keratinocytes. Furthermore, we compared the relative expression levels of CX3CL1 and CX3CR1 in MC, HaCaT, FB, as well as in human umbilical vein endothelial cells (HUVEC, known to express CX3CL1),^[^ ^38^, ^39^ ^]^ and human monocytic cells (THP‐1, known to express CX3CR1).^[^ ^39^, ^40^ ^]^ The results indicated that both CX3CL1 and CX3CR1 are relatively high expressed in MC cells (Figure 5b,c). The expression of CX3CL1 is susceptible to stimulation by external factors. Our previous research found that heat stress is an exposure factor causing skin pigmentation.^[^ ^8^ ^]^ Therefore, to determine whether CX3CL1 plays a role in heat stress‐induced melanogenesis, we subjected the MC and MNT1 cells to heat stress for 3 days to construct a cell model of heat stress‐induced melanogenesis (Figure 5d; Figure S7a, Supporting Information), and found that heat stress of 41°C could significantly up‐regulate the expression of CX3CL1 in MC and MNT1 cells (Figure 5e; Figure S7b,c, Supporting Information). In addition, heat stress can also induce the increase of CX3CL1 expression in human primary keratinocytes (KC) (Figure S7d, Supporting Information). Similarly, the expression of CX3CL1 also increased in the epidermal layer of the skin tissues exposed to heat stress for 5 days, as did the expression of CX3CR1 protein (Figure 5f; Figure S7e, Supporting Information). Furthermore, we collected a case of skin tissue with erythema ab igne, which is skin pigmentation caused by heat stress, and also observed relatively high expression of CX3CL1 and CX3CR1 in the epidermal layer of these tissues (Figure S7f, Supporting Information).

To further explore whether heat stress can promote melanogenesis by activating the CX3CL1‐CX3CR1 axis, we suppressed the expression of CX3CL1 in melanocytes and observed that the downregulation of CX3CL1 effectively counteracted the heat stress‐induced increase in melanin content (Figure 5g; Figure S7g, Supporting Information). Unsurprisingly, both the CX3CR1‐specific antagonist JMS‐17‐2 and CX3CR1 siRNAs (Figure S7h, Supporting Information) were able to reverse the increase in melanin content induced by heat stress (Figure 5h,i; Figure S7i, Supporting Information). Similarly, when human skin tissues treated with JMS‐17‐2 or CX3CR1 siRNAs were exposed to heat stress for 5 days, the same phenomenon was observed (Figure 5j,k).

### 2.6 Heat Stress Modulates the CX3CL1 Expression by Regulating WDR5‐Mediated H3K4me3 Modification

**Figure 6:**
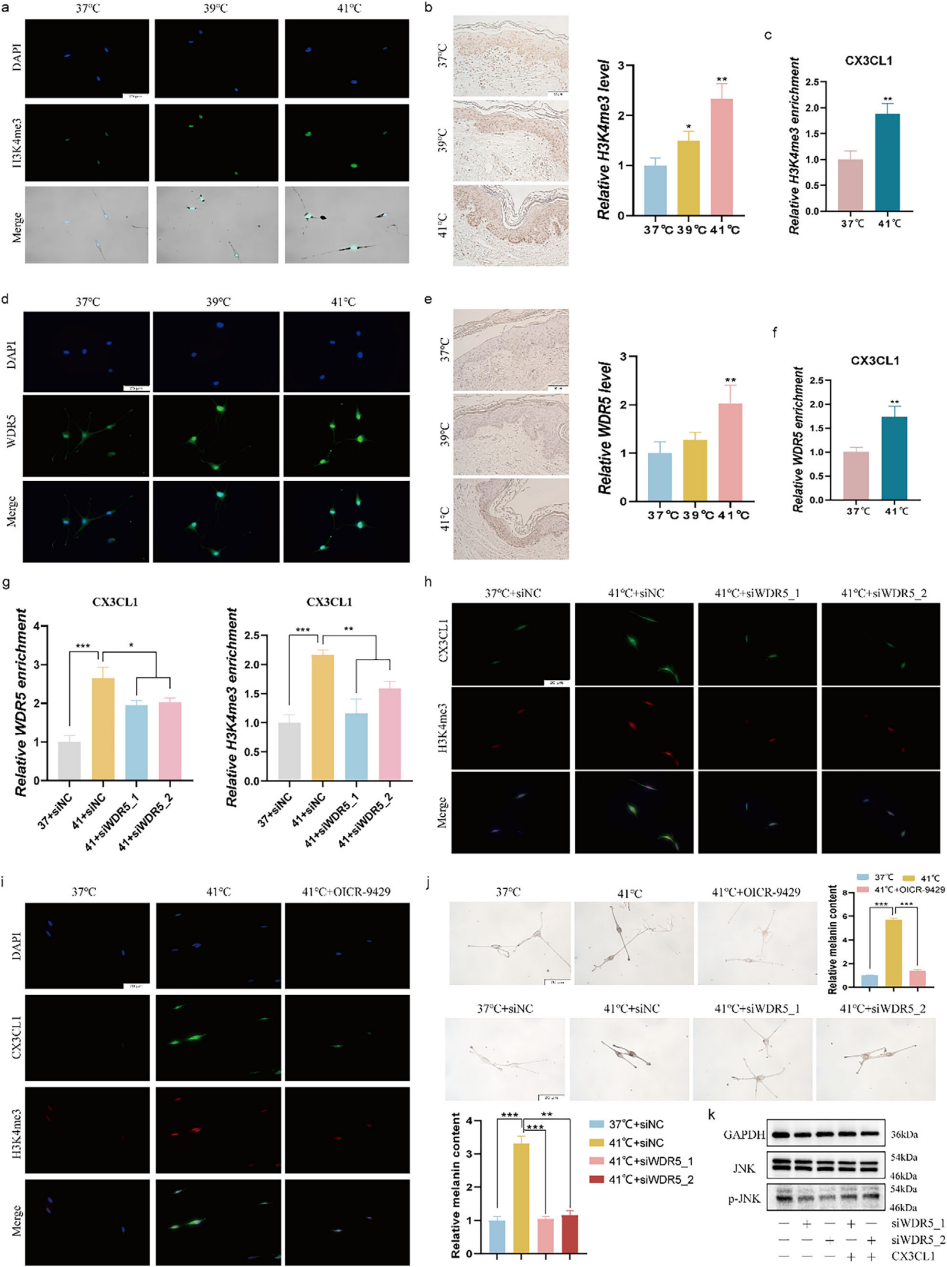
Heat stress modulates the CX3CL1 expression by regulating WDR5‐mediated H3K4me3 modification. a–f) After melanocytes or human skin tissue were exposed to heat stress for 3 or 5 days, respectively: the expression of H3K4me3 in MC cells and human skin tissues were detected by IF (a) or IHC (b), respectively; The enrichment level of H3K4me3 in the promoter region of CX3CL1 in MNT1 cells were detected by CUT&RUN‐qPCR (c); the expression of WDR5 in MC cells and human skin tissues were detected by IF (d) or IHC (e), respectively; The enrichment level of WDR5 in the promoter region of CX3CL1 in MNT1 cells were detected by CUT&RUN‐qPCR (f). g) After the MNT1 cells transfected with siWDR5 were exposed to heat stress for 3 days: the enrichment level of WDR5 and H3K4me3 in the promoter region of CX3CL1 in cells were detected by CUT&RUN‐qPCR. h–j) After the MC cells pretreated with OICR‐9429 for 30min or transfected with siWDR5 were exposed to heat stress for 3 days: the expression of H3K4me3 and CX3CL1 was detected by IF(h‐i), and the changes in melanin content was detected by Fontana‐Masson staining (j). k) MNT1 cells were transfected with siWDR5 for 48h and then treated with CX3CL1 for 4h, the level of the JNK signaling pathway was detected by Western blot. Scale=20µm. Statistical analysis was conducted via Unpaired Student's t‐test or one‐way ANOVA with multiple comparisons, mean ± SEM, n = 3 (^*^ *p* < 0.05, ^**^ *p* < 0.01, and ^***^ *p* < 0.001).

Histone modifications have a profound impact on the transcriptional regulation of chemokines.^[^ ^41^ ^]^ To further explore the mechanism by which heat stress induces CX3CL1 expression, we analyzed the Cistrome DB database and found that H3K4me3 is highly enriched in the CX3CL1 promoter region (Figure S8a, Supporting Information). After heat treatment of MC and MNT1 cells, the level of H3K4me3 increased (**Figure** **6**a; Figure S8b, Supporting Information), as well as in skin tissues (Figure 6b). Meanwhile, heat stress could upregulate the H3K4me3 enrichment level in the CX3CL1 promoter region of MNT1 cells (Figure 6c). This suggests that heat stress may selectively activate CX3CL1 through H3K4me3 modification. WDR5, ASH2L, RBBP5, and DPY30 are common subunits of histone methyltransferases complex.^[^ ^42^ ^]^ In heat‐treated MNT1 cells, the mRNA expression of WDR5 was significantly upregulated (Figure S8c, Supporting Information). Subsequently, we observed co‐localization of WDR5 and H3K4me3 within the cell nucleus (Figure S8d, Supporting Information). The protein levels of WDR5 were significantly upregulated in heat stressed‐MC and MNT1 cells (Figure 6d; Figure S8e, Supporting Information), as well as in heat stressed‐skin tissues (Figure 6e). And heat stress could increase the WDR5 enrichment level in the CX3CL1 promoter region of MNT1 cells (Figure 6f).

Furthermore, in order to explore the regulatory effect of WDR5 on H3K4me3 and CX3CL1 under heat stress conditions, we treated MNT1 cells with WDR5 siRNAs (Figure S9a,b, Supporting Information). The results demonstrated that suppression of WDR5 expression effectively reversed the heat stress‐induced upregulation of both H3K4me3 and WDR5 enrichment levels in the CX3CL1 promoter region (Figure 6g). Meanwhile, as shown in Figure 6h,i and Figure S9c,d (Supporting Information), both WDR5‐specific inhibitor ORIC‐9429 and silencing WDR5 expression antagonized the heat stress‐induced increase in CX3CL1 and H3K4me3 levels. This suggests that heat stress may activate H3K4me3 modification through WDR5, thereby promoting CX3CL1 expression. Additionally, to verify the regulatory role of the WDR5 in heat stress‐induced melanogenesis, we treated heat‐stressed melanocytes with ORIC‐9429 and siWDR5. The results showed that ORIC‐9429 and suppression of WDR5 expression effectively counteract the heat stress‐induced increase in melanin content (Figure 6j; Figure S9e, Supporting Information). As previously demonstrated, CX3CL1 enhances melanogenesis by upregulating the phosphorylation level of JNK (Figure 4). Consistent with these findings, WDR5 regulates melanogenesis through modulation of the JNK signaling pathway. Specifically, silencing the expression of WDR5 suppresses JNK phosphorylation, and this effect can be reversed upon exogenous administration of CX3CL1 (Figure 6k). However, JNK inhibitors have no significant effect on WDR5 expression (Figure S9f, Supporting Information).

### 2.7 MYC Mediates the Regulatory Effect of WDR5 on CX3CL1 Expression

**Figure 7:**
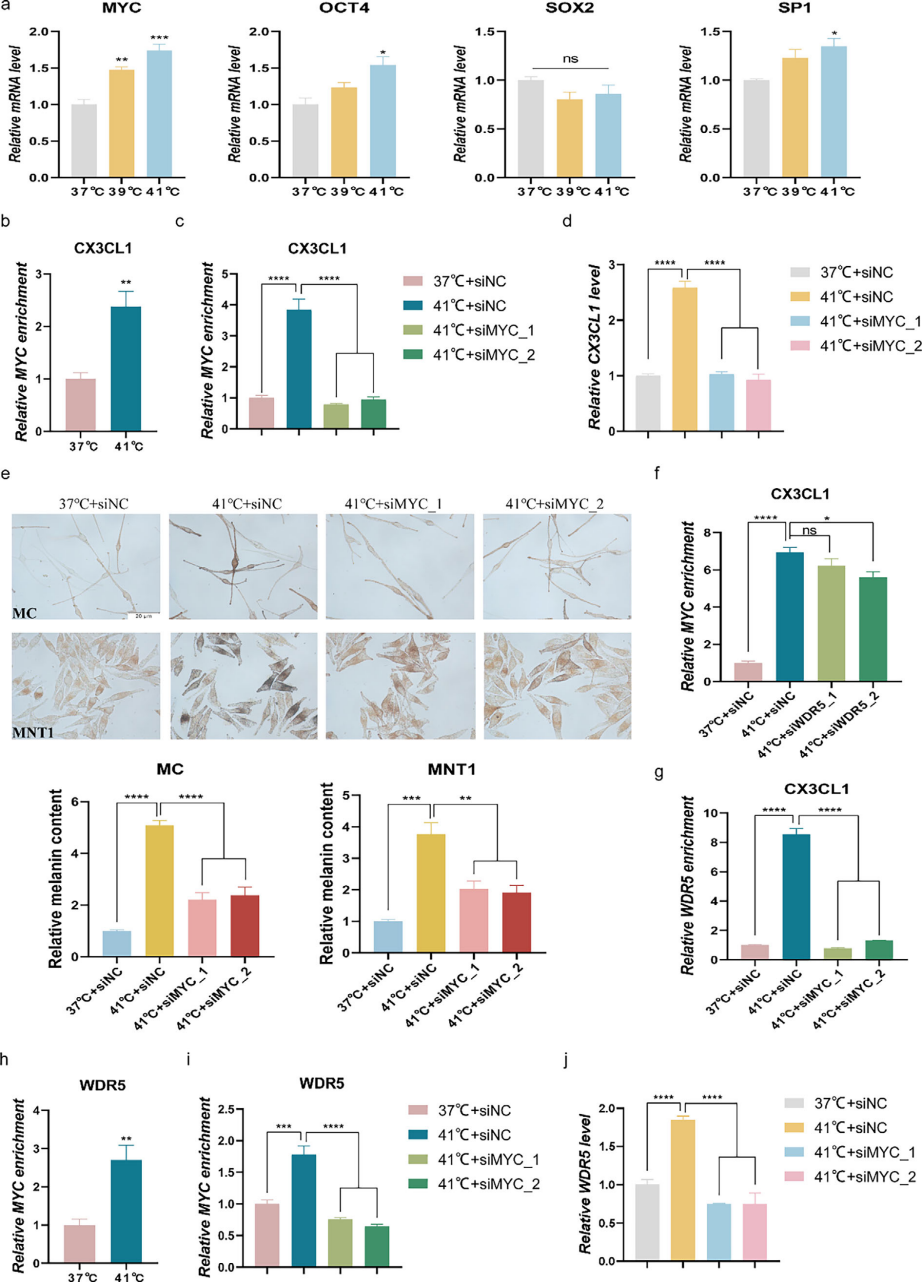
MYC mediates the regulatory effect of WDR5 on CX3CL1 expression. a,b) After MNT1 cells were exposed to heat stress for 3 days: the mRNA expression of MYC, OCT4, SOX2 and SP1 were detected by qRT‐PCR(a); The enrichment level of MYC in the promoter region of CX3CL1 were detected by CUT&RUN‐qPCR (b). c–e) After the MNT1 and MC cells transfected with siMYC were exposed to heat stress for 3 days: the enrichment level of MYC at the CX3CL1 promoter region in MNT1 cells was detected by CUT&RUN‐qPCR (c); the mRNA expression of CX3CL1 in MNT1 cells was detected by qRT‐PCR(d); and the changes in melanin content in both MC and MNT1 cells was detected by Fontana‐Masson staining, quantitative analysis was conducted using ImageJ (e). f) MNT1 cells transfected with siWDR5 were subjected to heat stress for 3 days, and the MYC enrichment level in the CX3CL1 promoter region was detected by CUT&RUN‐qPCR. g) MNT1 cells transfected with siMYC were subjected to heat stress for 3 days, and the WDR5 enrichment level in the CX3CL1 promoter region was detected by CUT&RUN‐qPCR. h) After MNT1 cells were exposed to heat stress for 3 days: the enrichment level of MYC in the promoter region of WDR5 were detected by CUT&RUN‐qPCR. i,j) After the MNT1 cells transfected with siMYC were exposed to heat stress for 3 days: the enrichment level of MYC in the promoter region of WDR5 were detected by CUT&RUN‐qPCR (i); the mRNA expression of WDR5 was detected by qRT‐PCR(j). Scale=20µm. Statistical analysis was conducted via Unpaired Student's t‐test or one‐way ANOVA with multiple comparisons, mean ± SEM, n = 3 (ns: no significant difference, ^*^ *p* < 0.05, ^**^ *p* < 0.01, ^***^ *p* < 0.001, and ^****^ *p* < 0.0001).

Our aforementioned research suggests that WDR5 can act as a critical epigenetic regulatory factor in the regulation of CX3CL1 expression. Existing studies have shown that WDR5 can serve as a key coactivator by interacting with transcription factors such as MYC, OCT4, and SP1 to influence transcriptional activity.^[^ ^43^, ^44^, ^45^ ^]^ To further explore the potential mechanisms, we analyzed the enrichment of transcription factors known to interact with WDR5 in the CX3CL1 promoter region using the Cistrome DB database. It was found that MYC, SOX2, OCT4, and SP1 were significantly enriched in the promoter region of CX3CL1 (Figure S10, Supporting Information). Subsequently, we detected the expression changes of the above transcription factors in MNT1 cells under heat stress. The results indicated that MYC was most significantly upregulated in the heat stressed‐cells (**Figure** **7**a). Furthermore, heat stress significantly increased the enrichment of MYC at the CX3CL1 promoter in MNT1 cells (Figure 7b). Subsequently, upon inhibition of MYC expression in melanocytes (Figure S11, Supporting Information), the heat stress‐induced increase in MYC enrichment at CX3CL1 promoter region was reversed (Figure 7c). Consistently, MYC knockdown abrogated the heat stress‐induced upregulation of CX3CL1 expression and melanogenesis (Figure 7d,e). These results indicate that MYC plays a crucial role in CX3CL1‐induced melanogenesis under heat stress conditions.

To further elucidate how the MYC‐WDR5 interaction influences CX3CL1 expression under heat stress, we transfected MNT1 cells with siWDR5 under heat stress conditions. We found that WDR5 knockdown slightly reversed the heat stress‐induced enrichment of MYC at the CX3CL1 promoter (Figure 7f). However, inhibiting MYC expression significantly abrogated the recruitment of WDR5 to the CX3CL1 promoter (Figure 7g), establishing that the WDR5 recruitment to the CX3CL1 promoter is MYC‐dependent. Furthermore, our study revealed that heat stress also significantly increased the enrichment of MYC at the promoter of the WDR5 in MNT1 cells (Figure 7h). Notably, MYC inhibition reversed both the enhanced MYC enrichment in the WDR5 promoter and the increased WDR5 mRNA expression induced by heat stress (Figure 7i,j).

## 3 Discussion

**Figure 8:**
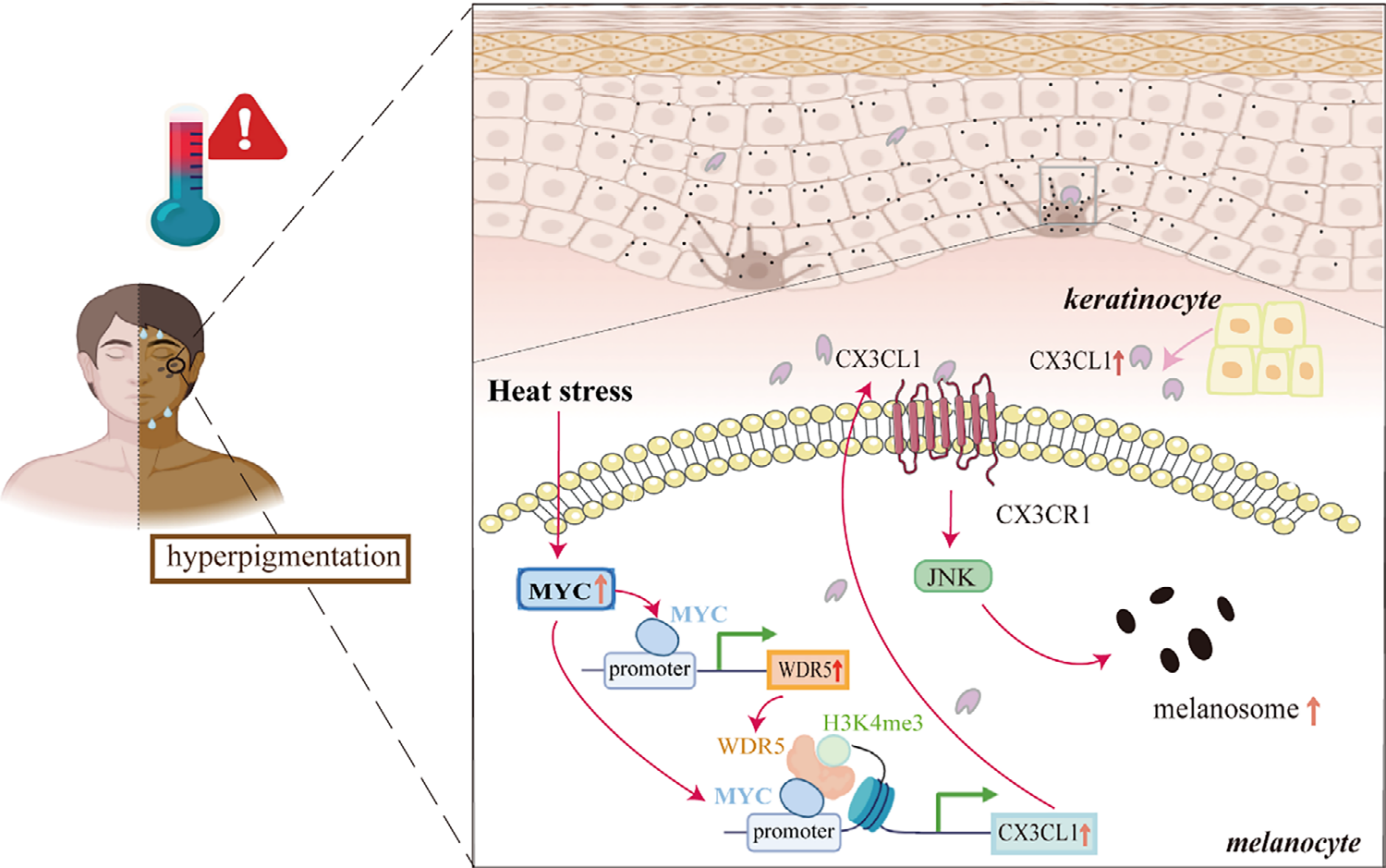
Schematic presentation of the mechanism by which Heat stress modulates MYC/WDR5‐mediated H3K4me3 modification to induce melanogenesis via activating CX3CL1/CX3CR1 axis.

In this study, we found that heat stress can induce the expression of CX3CL1 through MYC‐WDR5‐H3K4me3 axis, and the CX3CL1‐CX3CR1 axis can affect heat stress‐induced melanogenesis by regulating JNK pathways (**Figure** **8**).

Hyperpigmented dermatoses is secondary to inflammatory skin diseases, laser treatment, and external irritation, etc.^[^ ^46^, ^47^, ^48^ ^]^ Melasma is a common degenerative hyperpigmented skin disease, with an incidence of 30% in Asian women of childbearing age.^[^ ^49^ ^]^ In order to explore the relationship between CX3CL1 and melanogenesis, we analyzed the transcriptomes of the melasma skin tissue, and found that CX3CL1 was positively correlated with melanogenesis. Consistent with the bioinformatics data, pigmented nevi skin samples showed high expression levels of CX3CL1, particularly in regions with higher melanin content. Furthermore, CX3CL1 treatment increased the melanin content in cultured foreskin tissues and melanocytes. The inhibition of CX3CL1 was found to reverse the observed pro‐melanogenic effects. Furthermore, our data indicate that CX3CL1 can exert indirect effects on melanogenesis by modulating keratinocytes and fibroblasts, in addition to its direct action on melanocytes. This suggests a paracrine mechanism also contributes to its overall function. This study has focused on elucidating the direct regulatory effects of CX3CL1 on melanocytes and their underlying mechanisms. The potential influence of CX3CL1 on the paracrine functions of keratinocytes and fibroblasts remains an important avenue for future investigation.

Beyond modulating inflammatory mediators, CX3CL1 exhibits functional ambivalence. Its role is dictated by the surrounding context, demonstrating that its ultimate effect is determined by specific physiological and pathological conditions. For example, CX3CL1 can recruit and activate immune cells, trigger inflammatory cascades, and elevate levels of cytokines such as TNF‐α, IL‐6, and IL‐18, thereby contributing to processes including atherosclerosis and bone homeostasis.^[^ ^31^, ^32^ ^]^ In neuroscience, CX3CL1 has been shown to suppress the production of pro‐inflammatory cytokines (e.g., IL‐18, TNF‐α, and IL‐6) in microglia, highlighting its potential as a therapeutic target in neonatal prenatal stress and ischemic stroke.^[^ ^50^, ^51^, ^52^ ^]^ Since these cytokines are implicated in the complex regulation of melanogenesis, we investigated CX3CL1's effect by measuring a panel of pigmentation‐associated inflammatory mediators. In CX3CL1‐stimulated skin tissue, the expression of TNF (a known inhibitor of melanogenesis)^[^ ^15^ ^]^ was unchanged. In contrast, we observed significant upregulation of both suppressors (IL‐4,^[^ ^53^ ^]^, IL‐6 ^[^ ^33^ ^]^) and promoters (IL‐18,^[^ ^14^ ^]^ IL‐37^[^ ^11^ ^]^) of melanogenesis. This coordinated shift suggests that CX3CL1 remodels the inflammatory milieu, and the net outcome of these potentially antagonistic and synergistic interactions within this complex network is an overall promotion of melanogenesis.

CX3CR1 is the sole receptor for CX3CL1, and our results confirm for the first time that inhibiting CX3CR1 expression or using CX3CR1 antagonist JMS‐17‐2 reverses CX3CL1‐induced melanogenesis. Furthermore, we systematically evaluated the direct effect of JMS‐17‐2 on basal melanogenesis. Although no significant change was observed with a single low concentration (10nm) treatment, the concentration‐gradient experiments demonstrated that higher concentrations of JMS‐17‐2 (20nm and above) produced a concentration‐dependent inhibitory effect. This pharmacological phenotype aligns with the results observed in CX3CL1 knockdown. It is noteworthy that the concentration required to suppress basal melanogenesis is higher than that needed to reverse exogenous CX3CL1‐induced stimulation. This differential potency reveals a context‐dependent action of JMS‐17‐2: it potently antagonizes pathologically or experimentally elevated CX3CL1 signaling, while a stronger degree of pathway blockade is required to disrupt the robust basal autocrine activity that maintains physiological homeostasis.

Our studies have found that CX3CL1‐CX3CR1 axis mediates the development of depression by promoting JNK phosphorylation.^[^ ^54^ ^]^ Similarly, we found that the CX3CL1‐CX3CR1 axis promotes melanogenesis by activating the JNK signaling pathway. Moreover, we found that JNK activators can synergize with CX3CL1 to further enhance melanin production in melanocytes, whereas inhibition of the JNK signaling pathway in melanocytes attenuates the CX3CL1‐induced increase in melanin content. These findings suggested that CX3CL1‐CX3CR1 axis promote melanogenesis by activating the JNK pathway. It is noteworthy that as a core stress‐response pathway in cells, the JNK signaling pathway may exert diametrically opposite regulatory effects during melanogenesis. This discrepancy depends on various variables, including cell type, the nature of the stimulus, signal intensity and duration, as well as specific physiological or pathological contexts. Studies have confirmed that the JNK inhibitor SP600125 can effectively antagonize Nilotinib‐induced melanogenesis by blocking JNK phosphorylation in B16F0 cells.^[^ ^55^ ^]^ In Mel‐Ab cells, this inhibitor can significantly reverse Methylsulfonylmethane‐mediated enhancement of melanin synthesis.^[^ ^56^ ^]^ Furthermore, it can remarkably inhibit Flumequine‐induced melanogenesis in B16F10 cells and zebrafish larvae.^[^ ^57^ ^]^ Correspondingly, the JNK agonist anisomycin in B16F10 cells can reverse the suppression of melanogenesis mediated by Ganoderma lucidum polysaccharides.^[^ ^35^ ^]^ However, contrary phenomena have also been reported: SP600125 can attenuate the inhibitory effect of Paeonol on melanogenesis^[^ ^58^ ^]^; In Melan‐A cells, SP600125 significantly alleviate the inhibitory effects of OISEA^[^ ^59^ ^]^ (Oroxylum indicum Vent. seed extract) and NNFE^[^ ^60^ ^]^ (Nymphaea nouchali flower extract) on melanogenesis. Separately, anisomycin can counteract the melanogenesis promoted by Forskolin.^[^ ^61^ ^]^ These seemingly contradictory research findings collectively indicate that the regulation of melanogenesis by JNK is highly dependent on the specific signaling network environment. Notably, a review of the aforementioned literature reveals that changes in a single pathway are far from sufficient to fully explain the global regulatory mechanism of melanogenesis. In fact, in many of the above‐mentioned studies, the activation of other key signaling pathways such as cAMP/PKA often undergoes concurrent changes when the JNK pathway is regulated. This phenomenon suggests that the JNK pathway may engage in extensive crosstalk with other signaling pathways. These pathways work in concert to collectively determine the ultimate outcome of melanogenesis. Therefore, in the melanogenesis mediated by CX3CL1, CX3CL1 may simultaneously regulate other signaling pathways and work in synergy with the JNK pathway. As shown in Figure 1d, our analysis found that CX3CL1 may also exert regulatory effects on the cGMP‐PKG and PPAR signaling pathways. These two pathways have been reported to play significant regulatory roles in melanogenesis.^[^ ^62^, ^63^ ^]^ Therefore, in addition to the JNK pathway, CX3CL1 is likely to regulate melanogenesis through other pathways such as cGMP‐PKG and PPAR, and the specific mechanisms involved require further validation.

Studies have shown that CX3CL1 expression is sensitive to external stimuli.^[^ ^22^, ^23^, ^24^ ^]^ Heat stress are the common external irritants to the skin, and recent studies have shown that heat stress can induce skin pigmentation.^[^ ^8^, ^64^ ^]^ However, whether heat stress can stimulate the expression of CX3CL1 in skin cells remains unclear. In this study, we treated MC, MNT1, KC, and human skin tissue with heat stress, demonstrating that heat stress can upregulate CX3CL1 expression in the epidermis. Even more interestingly, the increase in melanin content induced by heat stress was also reversed upon inhibition of CX3CL1 or CX3CR1 expression, or following treatment with JMS‐17‐2, in both melanocytes and skin tissue. This suggests that heat stress may promote melanogenesis by inducing the expression of CX3CL1 of melanocytes. Furthermore, we also observed that heat stress can induce keratinocytes to express CX3CL1, suggesting that heat stress not only promotes melanogenesis by activating the autocrine effect of CX3CL1 in melanocytes but also activates keratinocytes to secrete CX3CL1, thereby stimulating melanocyte function through paracrine signaling.

Heat stress affects the epigenetic state of cells.^[^ ^65^, ^66^ ^]^ Histone modifications have a profound impact on the transcriptional regulation of chemokines.^[^ ^41^ ^]^ H3K4me3 is an important epigenetic marker, typically associated with gene activation.^[^ ^67^ ^]^ Through analysis of the Cistrome DB database, we found that H3K4me3 is highly enriched in the promoter region of CX3CL1. Moreover, studies have reported that MLL1 can induce CX3CL1 expression by upregulating H3K4me3 levels in the CX3CL1 promoter region, thereby contributing to the development of rheumatoid arthritis.^[^ ^41^ ^]^ Unsurprisingly, we also observed that heat stress upregulates H3K4me3 levels in the CX3CL1 promoter region in pigment cells, suggesting that heat stress may selectively activate CX3CL1 through H3K4me3 modification.

H3K4me3 is a reversible process dynamically regulated by histone methyltransferases (HMTs, such as SET1, MLL1‐5) and histone demethylases (KDMs, such as JHDM1A, JHDM1B, KDM5A). Among these, WDR5, ASH2L, RBBP5, and DPY30 are common subunits of histone methyltransferase complexes.^[^ ^42^, ^68^, ^69^ ^]^ Our study found that heat stress upregulates WDR5 expression in human skin tissues and melanocytes. Furthermore, after inhibiting WDR5 expression, the levels of H3K4me3 and CX3CL1 in melanocytes decreased, and the heat stress‐induced increase in melanin content was also reversed. Therefore, we speculate that heat stress may regulate H3K4me3 levels in the CX3CL1 promoter region and the expression of CX3CL1 by influencing the specific recruitment of WDR5 and ultimately promote melanogenesis. In addition, we found that WDR5 knockdown attenuated JNK pathway activation, an effect that was reversed upon supplementation with exogenous CX3CL1. These results indicate that WDR5 can influence the JNK signaling pathway by regulating the expression of CX3CL1.

In our subsequent studies, we found that the recruitment of WDR5 to the CX3CL1 promoter region under heat stress was MYC‐dependent. MYC is a super transcription factor that plays a core role in regulating cell growth, normal physiological processes and development.^[^ ^70^, ^71^ ^]^ While established theory emphasizes the fundamental role of WDR5 in recruiting MYC to chromatin,^[^ ^43^ ^]^ our study found that WDR5 knockout only slightly reversed the enrichment of MYC on the CX3CL1 promoter induced by heat stress. Instead, the inhibition of MYC expression significantly eliminated the recruitment of WDR5 to the CX3CL1 promoter. This suggests that, under heat stress conditions, the recruitment of WDR5 to the CX3CL1 promoter is MYC‐dependent. Additionally, heat stress can increase the enrichment of MYC in the WDR5 promoter region, thereby activating the transcription of WDR5. This study reveals that heat stress likely triggers the MYC‐WDR5‐H3K4me3 axis to drive CX3CL1 transcriptional activation. This finding not only elucidates the regulatory mechanism of CX3CL1 under heat stress but also provides a new model for understanding the dynamic regulation of MYC‐WDR5 interaction under stress conditions.

The pathogenesis of pigmentary skin disorders remains unclear, and their treatment has long been a clinical challenge. For hyper‐pigmentary disorders such as melasma, current prevention and treatment methods primarily include sun protection, tyrosinase inhibition, chemical peels, and laser therapy.^[^ ^72^, ^73^ ^]^ For hypo‐pigmentary disorders such as vitiligo, the treatment methods primarily include glucocorticoids and calcineurin inhibitors combined with phototherapy, JAK inhibitors, surgical treatments.^[^ ^74^ ^]^ However, issues such as inconsistent efficacy, high costs, frequent recurrence, and significant side effects persist, imposing a heavy burden on individuals, families, and society. Therefore, there is an urgent need to explore new mechanisms influencing skin pigmentation and develop more convenient and effective treatments. Research indicates that localized heat stress significantly contributes to the development of melasma.^[^ ^10^ ^]^ Our findings suggest that reducing heat exposure or developing topical CX3CL1 inhibitors could be a novel approach to preventing and treating melasma. Additionally, studies have shown that fibroblasts around wounds secrete CXCL12, which recruits CXCR4‐expressing melanocyte (MC) precursors or CXCR7‐expressing mature MCs from the hair follicle bulge or lesion periphery to migrate into vitiligo lesions and stimulate repigmentation.^[^ ^75^ ^]^ Could CX3CL1, secreted by melanocytes and keratinocytes under heat stress, play a similar role in promoting vitiligo repigmentation? Other studies have also demonstrated that heat enhances the melanogenesis‐promoting effects of UVB.^[^ ^64^ ^]^ Therefore, might combining heat exposure with phototherapy enhance vitiligo repigmentation further? These hypotheses, of course, require further experimental and clinical validation.

In conclusion, our findings elucidate a novel mechanism whereby heat stress promotes melanogenesis through MYC/WDR5/H3K4me3‐mediated epigenetic regulation of CX3CL1, which subsequently activates the CX3CR1‐JNK signaling pathway. This mechanistic insight may offer innovative therapeutic strategies and molecular targets for intervening in pathological pigmentation processes.

## 4 Experimental Section

### Reagents

CX3CL1 was purchased from Cloud‐Clone Corp (Wuhan, China). Four percent neutral paraformaldehyde and Cell Counting Kit‐8 (CCK8) were purchased from Biosharp (Hefei, China), and Fontana‐Masson Stain kit was from G‐ClONE (Beijing, China). Sodium deoxycholate and L‐dopa were purchased from Solarbio (Beijing, China), and TritonX‐100 from Sigma–Aldrich. Dulbecco's modified Eagle medium (DMEM), RPMI‐1640, Medium 254 (#M‐254‐500), Keratinocytes Serum Free Medium (Defined K‐SFM), human melanocyte growth supplement (HMGS), human keratinocytes growth supplement (HKGS), penicillin‐streptomycin (P/S), non‐essential amino acids (NEAA) and penicillin/streptomycin/amphotericin B were purchased from Gibco (Maryland, USA). JMS‐17‐2(#B8536) was purchased from APExBIO (Houston, USA). JNK‐IN‐18(HY‐13319), SP600125(HY‐12041), Anisomycin(HY‐18982) and OICR‐9429 (HY‐16993) were purchased from MedChemExpress (New Jersey, USA). The primary antibodies and their suppliers are as follows: GAPDH (#2118, Cell Signaling Technology), H3K4me3(#9751, Cell Signaling Technology), H3 (AF0009, Beyotime), TYR (R381782, ZENBIO), MITF (R24980, ZENBIO), TYRP1 (R382326, ZENBIO), DCT (R821374, ZENBIO and ER65221, HUABIO), PMEL17(#H1219, Santa Cruz and bsm‐61122R, Bioss), P38 (#8690, Cell Signaling Technology), p‐P38 (#4511, Cell Signaling Technology), JNK (#9252, Cell Signaling Technology), p‐JNK (#4668, Cell Signaling Technology), ERK (#4695, Cell Signaling Technology), p‐ERK (#4370, Cell Signaling Technology), CX3CL1 (bs‐0811R, bs‐23140R Bioss), CX3CR1 (bs‐1728R, Bioss), WDR5 (15544‐1‐AP, proteintech) and MYC (#18583, Cell Signaling Technology).

### Cell Culture

After approval by the Ethics Committee of the Third Xiangya Hospital of Central South University (No.24910), the human epidermal melanocytes (MC), human epidermal keratinocytes (KC) and human dermal fibroblasts (FB) were extracted from adolescent foreskin tissues donated after circumcision, and cultured in Medium 254 or Defined K‐SFM or DMEM supplemented with 1% HMGS or 1% HKGS and 1%penicillin/streptomycin/amphotericin B, respectively. The melanin‐rich human melanoma cell line MNT1,^[^ ^25^ ^]^ A2058^[^ ^26^ ^]^ and MEWO^[^ ^27^ ^]^ were purchased from Meisen (Wuhan, China) and Cyagen (Shanghai, China), respectively. Human immortalized keratinocytes HaCaT were purchased from Otwo Biotech (Shenzhen, China). The human monocytic cells THP‐1 was purchased from Fenghui Biotechnology (Hunan, China). Human umbilical vein endothelial cells HUVEC were purchased from ATCC (Mansas, Virginia, USA). PIG1 cells are a gift from Dr. Caroline Le Poole of Loyola University Chicago. Cells were cultured in DMEM or 1640 supplemented with 10% or 20% FBS (Meisen,Vivacell) and 1% P/S. All cells were incubated at 37 °C with 5% CO2.

### Cell and Human Skin Tissue Transfection

An appropriate number of MC or MNT1 cells were seeded in 6‐ or 12‐well plates. Depending on experimental requirements, transfection was performed when cell confluence reached either 20%–30% or 50–60%. The knockdown sequences for CX3CL1 (small interfering RNA, siCX3CL1), CX3CR1 (small interfering RNA, siCX3CR1), WDR5 (small interfering RNA, siWDR5) and MYC (small interfering RNA, siMYC) were synthesized by GenePharma (Shanghai, China). Cell transfection was carried out using the GP‐transfect‐Mate reagent (GenePharma). Subsequent experiments were conducted 24 or 48 h after transfection.

The knockdown sequences for CX3CR1(small interfering RNA, si_CX3CR1) were also synthesized by GenePharma. The isolated human skin foreskin tissue was cultured for 5 days, with local injection at a depth between the dermis and epidermis of 30µL (2.5 nmol) si_CX3CR1 administered on day 1 and day 3. The human skin tissues were cultured using the previously described methodology.^[^ ^11^ ^]^ All knockdown sequences are shown in Table S1 (Supporting Information).

### Heat Stress Treatment

Inoculate an appropriate amount of MC, MNT1, or KC cells into 6‐well or 12‐well plates. According to experimental requirements, when cell confluence reaches 20%–30% or 50–60%, exposed the cells to heat stress (37°C, 39°C, 41°C) for 30 min daily for 3 days. Human foreskin samples were subjected to heat stress for 40 min daily for 5 days.

### Cell Viability Assay

Cell viability was detected by the CCK8 assay. Briefly, the MNT1 cells were seeded in 96‐well plates at the density of 2000–3000 cells/well and treated with different concentrations of CX3CL1 for 24 or 48 h. At the stipulated time points, 10 µL CCK8 solution was added to each well and the cells were incubated for 2h. The absorbance was measured at 450 nm using a microplate reader (PerkinElmer EnVision xcite, UK).

### Fontana‐Masson Staining (MF)

Cultured cells were fixed with 4% neutral paraformaldehyde and stained with Fontana ammonia‐silver solution for 30 min at 56 °C in a water bath. The paraffin sections, were stained with ammonia‐silver solution and then counterstained with neutral red for 5 min. The melanin granules were observed under an inverted microscope (Olympus X71, Japan).

### Melanin Quantification

Cells stimulated with CX3CL1 for 48h were harvested, and resuspended in 1mL 10% DMSO (diluted with 1mm NaOH) at the density of 10^6^ cells/ml. After incubating at 80°C for 1h to dissolve the melanin, the mixture was transferred to a 96‐well plate and the absorbance at 470 nm was measured using a multi‐template reader.

### Measurement of TYR Activity

The cells were harvested after 48h of stimulation with CX3CL1, washed with PBS, and resuspended in 500 µL 0.5% sodium deoxycholate at the density of 2 × 10^6^ cells mL^−1^. The cells were lysed by incubating at 4 °C for 15 min, and then at 37 °C for 10 min. After adding 1 mL of 0.1% L‐DOPA, the lysates were incubated at 37 °C for 30 min, and 200 µL aliquots was dispensed in each well of 96‐well plate. The absorbance at 475 nm was measured using a multi‐template reader.

### Quantitative Real‐Time PCR (qPCR)

Total RNA was extracted from the suitably treated cells using Fast total RNA extraction kit (FASTAGEN, Shanghai, China), and reverse transcribed to cDNA using Reverse transcription kit (TransGen, China). The qPCR reactions were performed using the qPCR Mix (TransGen, China) on Roche LightCycler480II (Basel, Switzerland) with GAPDH as the internal reference. The primer sequences are shown in Table S2 (Supporting Information).

### Western Blot

The suitably treated cells were harvested, and lysed by homogenizing in the RIPA lysis buffer (Thermo Fisher) supplemented with 1% Protease Inhibitor and 1% Phosphatase Inhibitor cocktails (Roche). Equal amounts of protein were separated by 10% or 12.5% SDS‐PAGE (YEASON) and transferred to PVDF membranes (0.2 µm). After blocking with 20% rapid sealing fluid (Biosharp), the membranes were incubated overnight with primary antibodies (1:1000 or 1:2000) at 4 °C. The membranes were washed thrice with TBST, and then incubated with goat anti‐rabbit or anti‐mouse secondary antibody (1:10 000, LI‐COR Biosciences) for 1 h at 37 °C. The signal intensities of the protein bands were measured using the Odyssey CLx Imaging System (Li‐COR Biosciences).

### Immunohistochemistry (IHC)

Skin samples were collected at the Dermatology Department of the Third Xiangya Hospital of Central South University after obtaining approval form the Ethics Committee (No. 24910). After dewaxing, antigen repair, endogenous peroxidase inactivation, and serum blocking, the paraffin sections were incubated overnight with primary antibodies (1:200 diluted for CX3CL1, CX3CR1, and WDR5 antibodies, and 1:1000 for H3K4me3 antibodies) at 4 °C. Following incubation with reaction enhancing solution for 20 min and the secondary antibodies for 40 min, the sections were stained with DAB (ZLI‐9018, ZSGB‐BIO). The sections were observed under an inverted fluorescence microscope (Zeiss, China). Immunohistochemistry was performed using the PV‐9000 universal two‐step detection kit (ZSGB‐BIO, China).

### Immunofluorescence (IF)

The suitably treated cells were fixed with 4% neutral paraformaldehyde for 15 min, permeabilized with 0.5% Triton X‐100 for 10 min and blocked with 5% BSA for 1h. After incubating overnight with primary antibody (1:200 diluted for PMEL17, CX3CL1, CX3CR1, and WDR5 antibodies, and 1:400 for JNK, p‐JNK, H3K4me3, and MYC antibodies) at 4 °C, the cells were incubated with by donkey anti‐mouse or anti‐rabbit Alexa Fluor 488 or 594 (Beyotime Biotech; 1:400) at 37 °C for 1 h. In addition, for the simultaneous detection of two antibodies of the same species, we used a two‐label three‐color multiplex fluorescence staining kit (AiFang biological, China). The cells were counterstained with DAPI for 5 min, and the fluorescence intensity was observed under an inverted microscope.

### CUT&RUN‐qPCR

Ten thousand suitably treated cells were collected and the Hyperactive pG‐MNase CUT&RUN Kit (Vazyme, China) was used to detect the binding of the CX3CL1 promoter region with H3K4me3, WDR5, and MYC, as well as the binding of the WDR5 promoter region with MYC. The CT value was corrected by “spike in” negative primers. CX3CL1 primer sequence 1 (for detecting the binding of the CX3CL1 promoter region with H3K4me3): Forward: CACTCTGATGCTGTGGATAACCTG, Reverse: AGGGTCTGCTGAAAGAAGGATTG. CX3CL1 primer sequence 2 (for detecting the binding of the CX3CL1 promoter region with WDR5): Forward: GAGAAGCCCAGGGACACAAA, Reverse: ATAATGGGGTTGGCTGCCTC. CX3CL1 primer sequence 3 (for detecting the binding of the CX3CL1 promoter region with MYC): Forward: GCCCACTTAGGATCAAGGGC, Reverse: CTGGGTACAAAGGCCTCCTC. WDR5 primer sequence (for detecting the binding of the WDR5 promoter region with MYC): Forward: CTGCACCAGTCAAATTCGGG, Reverse: CTTTAAAGGGACAGCACGGG.

### Statistical Analysis

Transcriptomic data was processed and visualized using R (version 4.0.3). The experimental data was visualized using GraphPad (version 8.0.2). Unpaired Student's t‐test was used to compare two groups, and one‐way analysis of variance (ANOVA) was used for multiple group comparisons. *p* < 0.05 was considered statistically significant.

## Conflict Of Interest

The authors declare no conflict of interest.

## Author Contributions

Conception of manuscript: Q.Z. and Y.Z. conceived and designed this study. Y.Z. performed the experiments. Y.Z. and B.Y. analyzed and visualized the data. L.J. and C.F. collected the human skin tissue. Y.Z. wrote the manuscript. J.H., J.C., and Q.Z. provided the funding support. All authors reviewed and edited the manuscript.

## Supporting information



Supporting Information

## Data Availability

The data that support the findings of this study are available from the corresponding author upon reasonable request.
