# Supplementary material for: Heat Stress Modulates WDR5‐Mediated H3K4me3 Modification to Induce Melanogenesis via Activating CX3CL1/CX3CR1 Axis
Source: Adv Sci (Weinh). 2025 Nov 20;13(7):e10164. doi: 10.1002/advs.202510164 (PMC12866689; doi:10.1002/advs.202510164)
Supplement: Supplementary file 1 — Supporting Information [file ADVS-13-e10164-s001.pdf]

Heat stress modulates WDR5-mediated H3K4me3 modification to induce melanogenesis via activating CX3CL1/CX3CR1 axis

Yushan Zhang, Ling Jiang, Yibo Hu, Chuhan Fu, Jinhua Huang, Jing Chen, and Qinghai Zeng

Supplementary Tables

Supplementary Table 1. Information of knockdown sequences:

Gene	Sense (5'-3')	Antisense (5'-3')
si_CX3CR1_1	GCUCAGUCCACGUGAUUUTT	AAAUC AACGUGGACUGAGCTT
si_CX3CR1_2	CCUGAAUCCUCUCAUCUAUTT	AUAGAUGAGAGGAICAGGTT
si_WDR5 _1	GCUGGGAAUAUCCGAUGUATT	UACAUCGGAUAUUCCCAGCTT
si_WDR5 _2	UCUGGAACCUUCAGACGAATT	UUCGUCUGAAGGUUCCAGATT
si_MYC_1	GCUUGUACCUGCAGGAUCUTT	AGAUCCUGCAGGUACAAGCTT
si_MYC_2	GGCGAACACACAACGUCUUTT	AAGACGUUGUGUGUUCGCCTT

Supplementary Table 2. Information of primer sequences:

Gene	Forward	Reverse
GAPDH	CTCTGCTCCTCCTGTTCGAC	GCCCAATACGACCAAATCC
MITF	AAATACGTTGCCTGTCTCGG	TGTTGGGAAGGTTGGCTGGA
TYR	TCAGCCCAGCATCATTCTTC	GGCATCCGCTATCCCAGTAA
TYRP1	ACCAGAGGGTTCTCATAGTCAG	TTCTCAAATTGTGGCGTGTT
DCT	GGGCAGCGAGACCAGACGAT	TTGGCAATTTCATGCTGTTTCTTC
CX3CL1	ACGGTGTGACGAAATGCAAC	GCTGTCTCGTCTCCAAGATGAT
CX3CR1	CAGGGAAAGGTGGCCAAACA	CCATGGTGAAGGCCTCTAGT
WDR5	ATCTCGCTCAACAGACTGCC	TCACAGGTGTAGGCTTGCTC
ASH2L	TGTACAAGAGCTGCACGGTT	CCCATGTCACTCATAGGGCG
RBBP5	CTCGAGTTGCTGGAGTCCTT	GGTGCAAGTCAAAGCCATGC
DPY30	GCGGGTAATCAGCTCCCTT	CCCGGATACCAGTCTGGGC
MYC	TCTCTCCGTCCTCGGATTCT	TTCTTGTTCCTCCTCAGAGTCG
SOX2	ATGGACAGTTACGCGCACAT	CGAGCTGGTCATGGAGTTGT
OCT4	ACCCACACTGCAGCAGATCA	CCACACTCGGACCACATCC
SP1	GCCACCATGAGCGACCAAG	GTGAGGTCAAGCTCACCTGT

Supplementary Table 3. Information of GEO datasets:

GEO accession	Platform	Size	Source tissue	Experiment type
GSE72140	GPL570	48	Human skin	Array
GSE150672	GPL18573	438	Human skin	scRNA sequencing

Supplementary Figures and Legends

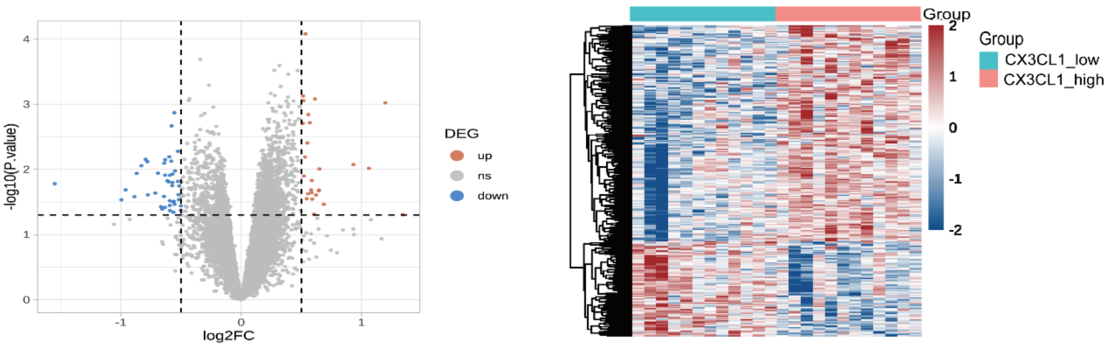

Figure S1. Visualization of DEGs grouped by CX3CL1 expression in GSE72140 dataset. (The left picture is a volcanic plot: red represents up-regulated genes and blue represents down-regulated genes. The right picture is a heat map: red represents the CX3CL1^{high} group and blue represents the CX3CL1^{low} group.)

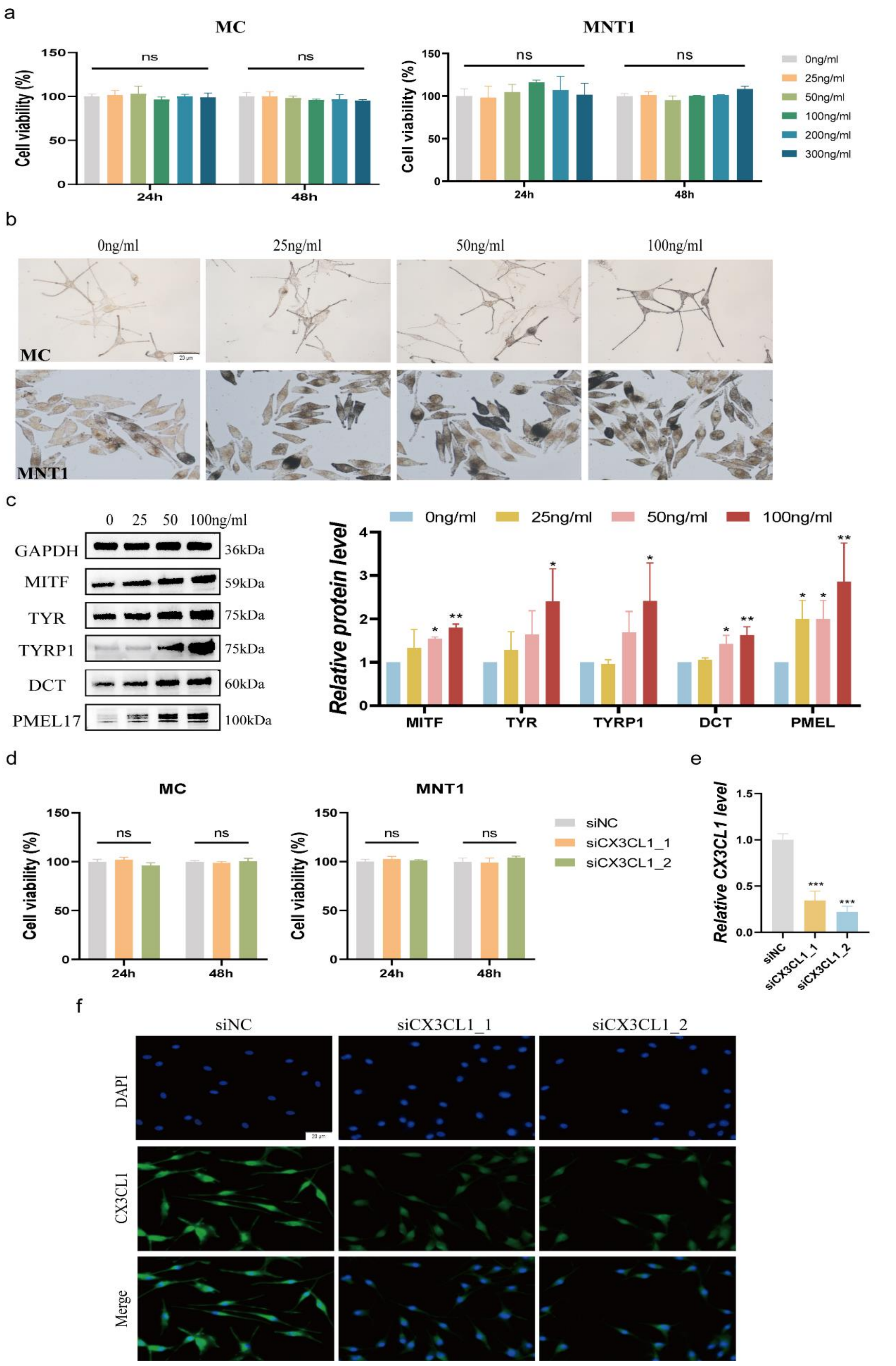

Figure S2. CX3CL1 can directly promote melanogenesis in melanocytes. (a) After

MC and MNT1 cells were treated with different concentrations of CX3CL1 for 24 or 48h, the viability of cells was detected by the CCK8 method. (b) MNT1 or MC cells treated with CX3CL1 for 24h, representative images of cells stained with Fontana-Masson showing melanin granules. (c) MNT1 cells treated with CX3CL1 for 48h, western blot showing the protein expression of key melanogenesis-related genes and PMEL17, and quantitative analysis was conducted using ImageJ. (d-f) si_CX3CL1 was transfected into MC and MNT1 cells: after 24 or 48h, the cell viability was detected by CCK8 method (d), the expression of CX3CL1 in MNT1 cells was detected by qRT-PCR after 24h (e), and the expression of CX3CL1 in MC cells was detected by IF after 48h (f). Scale=20μm. Statistical analysis was conducted via a one-way ANOVA with multiple comparisons, mean ± SEM, n = 3 (ns: no significant difference, *$P<0.05$, **$P<0.01$, ***$P<0.001$).

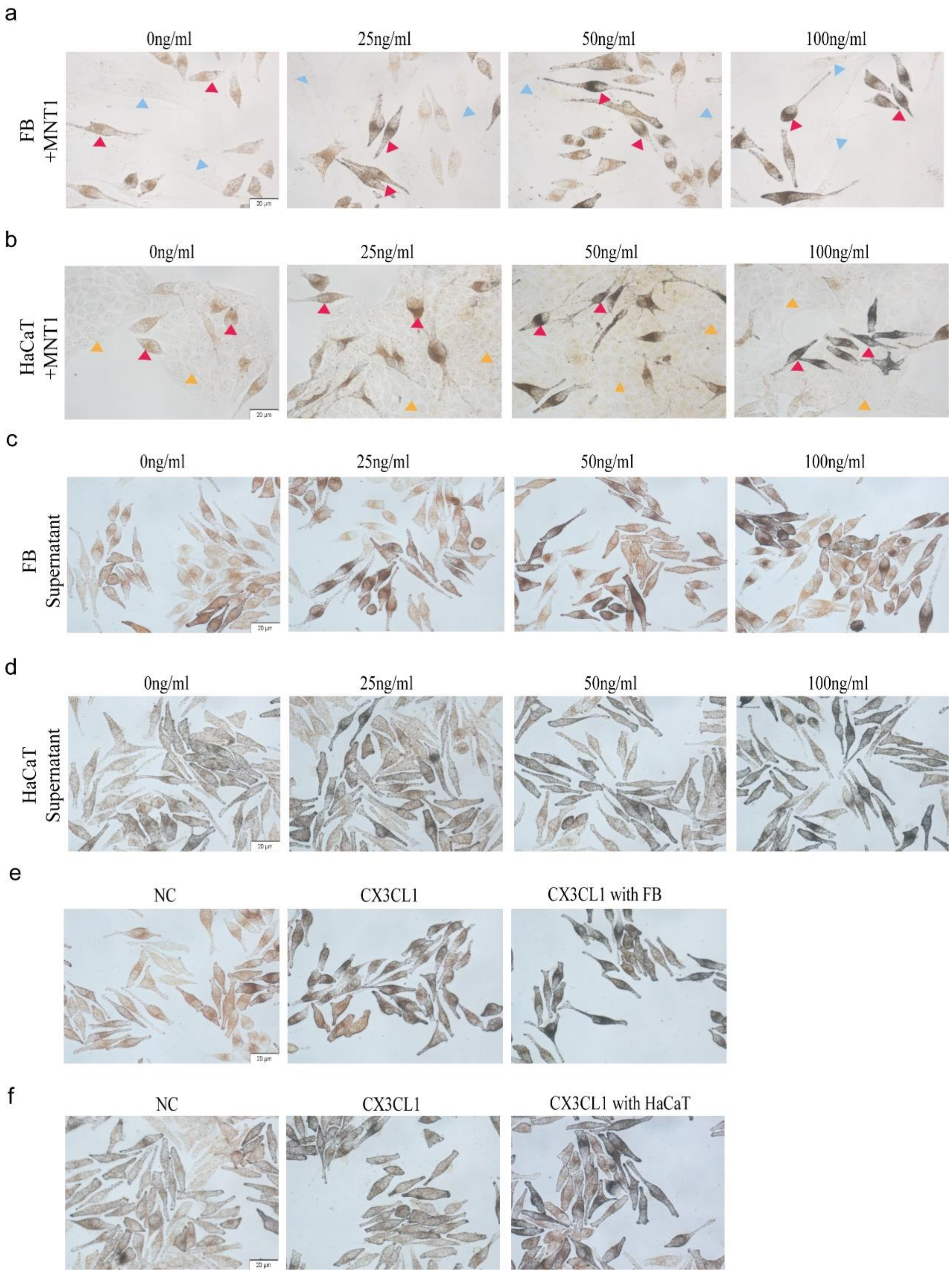

Figure S3. CX3CL1 can promote melanogenesis through the paracrine pathway. (a-b) CX3CL1 treated the co-culture system of MNT1 with FB (a) or HaCaT (b) for 24h, representative images of cells stained with Fontana-Masson showing melanin granules (The red arrow points to MNT1, the blue arrow points to FB, and the yellow arrow points to HaCaT). (c-d) After treating FB (c) and HaCaT (d) cells with CX3CL1 for 24h, the medium was replaced with complete medium without CX3CL1, and the

cells were cultured for an additional 24h. The supernatant was then collected and treated MNT1 cells for 24h, representative images of cells stained with Fontana-Masson showing melanin granules. (e-f) Conditioned media were prepared by incubating 100 ng/mL CX3CL1 with FB (e) or HaCaT (f) cells, or in the absence of cells, for 24h. MNT1 cells were then treated with these conditioned media for another 24h, representative images of cells stained with Fontana-Masson showing melanin granules. Scale=20μm.

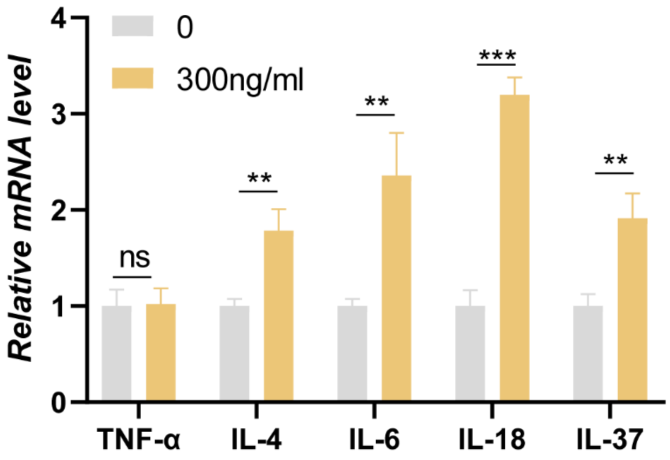

Figure S4. The regulation of CX3CL1 on the expression of other inflammatory factors. After CX3CL1 treatment of human skin tissue for 5 days, the expression of inflammatory factors in the tissue was detected by qRT-PCR. Statistical analysis was conducted via Unpaired Student's t-test, mean ± SEM, n = 3 (ns: no significant difference, **P<0.01, ***P<0.001).

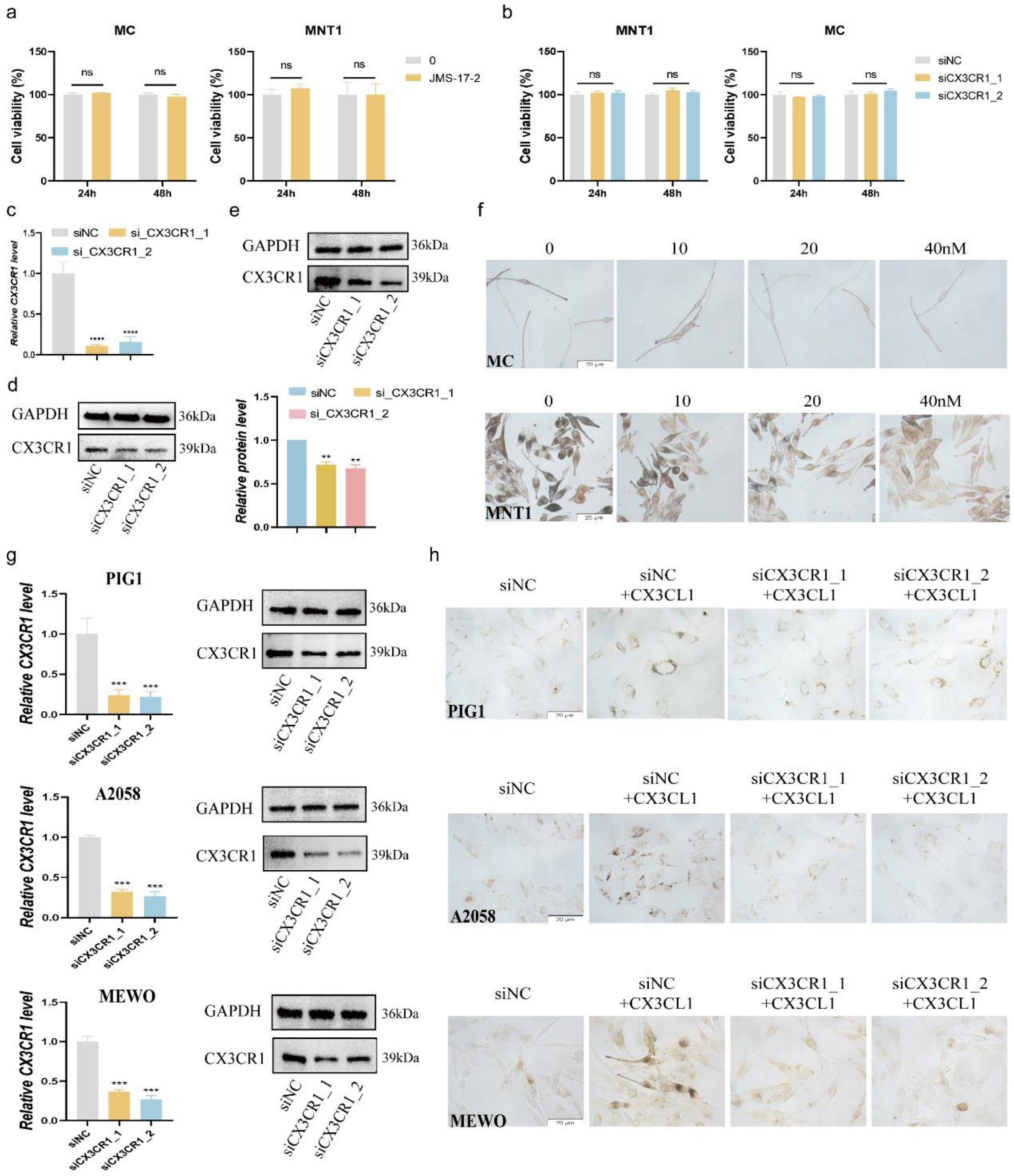

Figure S5. CX3CL1 regulates melanogenesis via the receptor CX3CR1. (a-b) After MC and MNT1 cells were treated with JMS-17-2 (10nM) or siCX3CR1 for 24 or 48h, the viability of cells was detected by the CCK8 method. (c-d) MNT1 cells were transfected with siCX3CR1, after 24h, the expression of CX3CR1 in cells was detected by qRT-PCR(c); after 48h, the expression of CX3CR1 in cells was detected by western blot(d). (e) Five days after local injection of si_CX3CR1 at the depth between the dermis and epidermis of isolated human foreskin tissue, the expression of CX3CR1 was detected by western blot. (f) MC and MNT1 cells were treated with JMS-17-2 for 24h, and the changes in melanin content were observed by Fontana-Masson staining. (g) PIG1, A2058 and MEWO cells were transfected with siCX3CR1, after 24h, the

expression of CX3CR1 was detected by qRT-PCR; and 48h later, the expression of CX3CR1 was detected by western blot. (h) After siCX3CR1-transfected cells were treated with CX3CL1 (100 ng/mL) for 24h, the changes in melanin content in the cells were detected by Fontana-Masson staining. Scale=20μm. Statistical analysis was conducted via Unpaired Student's t-test or one-way ANOVA with multiple comparisons, mean ± SEM, n = 3 (ns: no significant difference, ***P<0.001, ****P<0.0001).

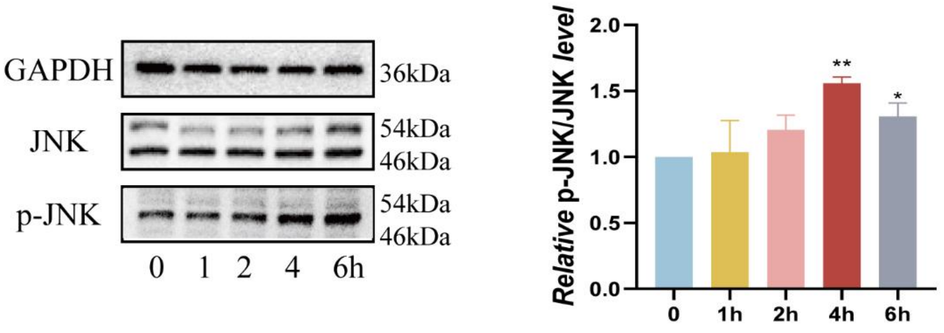

Figure S6. CX3CL1 upregulates the phosphorylation level of JNK. After MNT1 cells were treated with CX3CL1, proteins were collected at different time points, and the protein levels of the JNK signaling pathway were detected by western blot. Quantitative analysis was conducted using ImageJ. Statistical analysis was conducted via a one-way ANOVA with multiple comparisons, mean ± SEM, n = 3 (*P<0.05, **P<0.01).

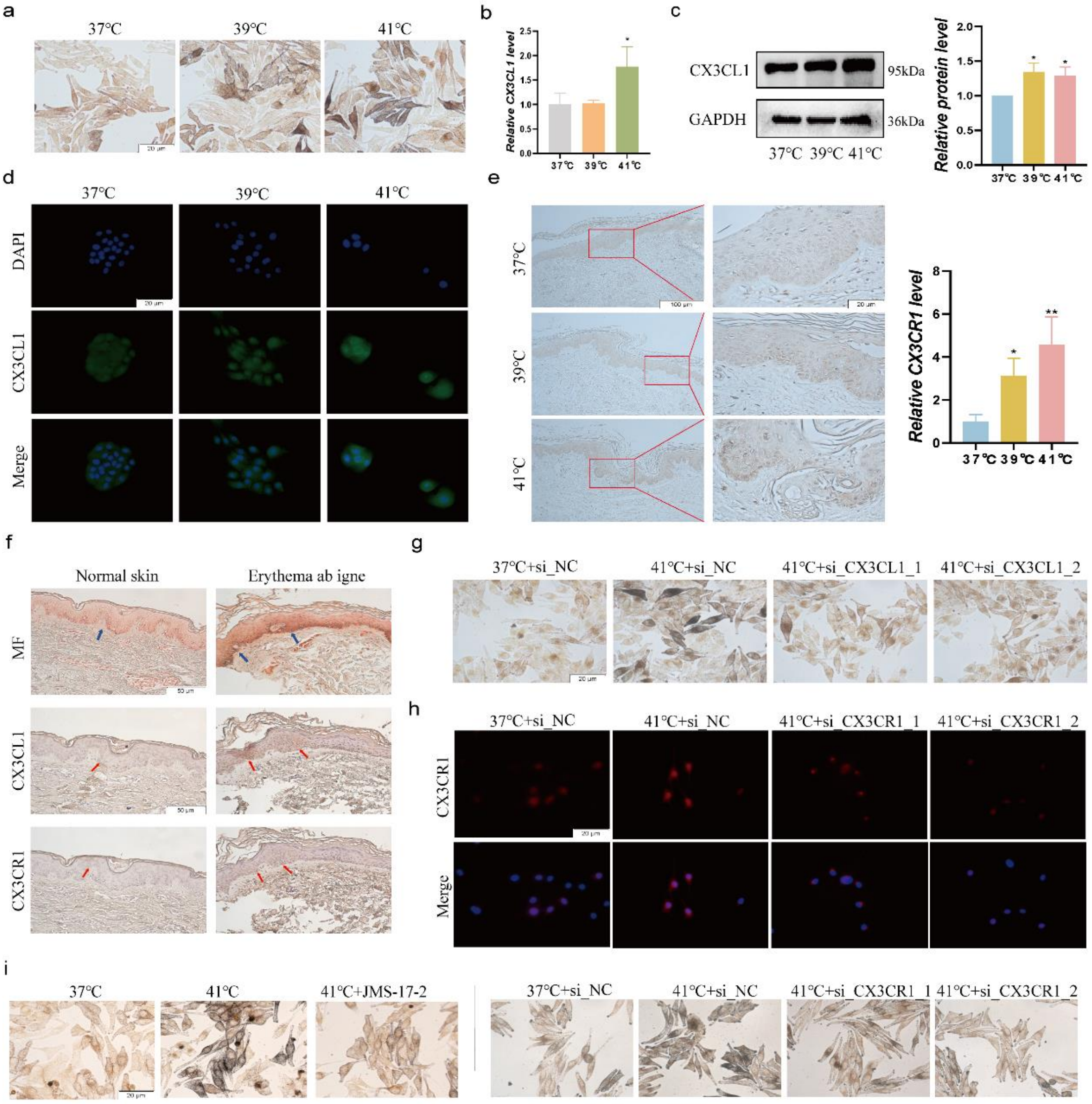

Figure S7. Heat stress can stimulate the expression of CX3CL1. (a-c) After MNT1 cells were exposed to heat stress for 3 days: Fontana-Masson staining was used to detect the melanin content (a) (Scale=20μm); qRT-PCR (b) and Western blot (c) was used to detect the expression of CX3CL1. (d) After KC cells were exposed to heat stress for 3 days, the expression of CX3CL1 was detected by IF (Scale=20μm). (e) After human foreskin tissues were exposed to heat stress for 5 days, the expression of CX3CR1 was detected by IHC (Scale=20μm and 100μm), and quantitative analysis was conducted using ImageJ. (f) Representative images of tissue sections stained with Fontana-Masson and IHC showing melanin granules and CX3CL1 or CX3CR1 protein expression in the skin tissue (Scale=50μm). (g) MNT1 cells transfected with siCX3CL1 were exposed to heat stress for 3 days, the changes in melanin content were detected by Fontana-Masson staining (Scale=20μm). (h) MC cells transfected with siCX3CR1 were exposed to heat stress for 3 days, the expression of CX3CR1 was detected by IF (Scale=20μm). (i) MNT1 cells pretreated with JMS-17-2 for 30min or transfected with siCX3CR1 were exposed to heat stress for 3 days, the changes in melanin content were detected by

Fontana-Masson staining (Scale=20μm). Statistical analysis was conducted via a one-way ANOVA with multiple comparisons, mean ± SEM, n = 3 (*P<0.05, **P<0.01).

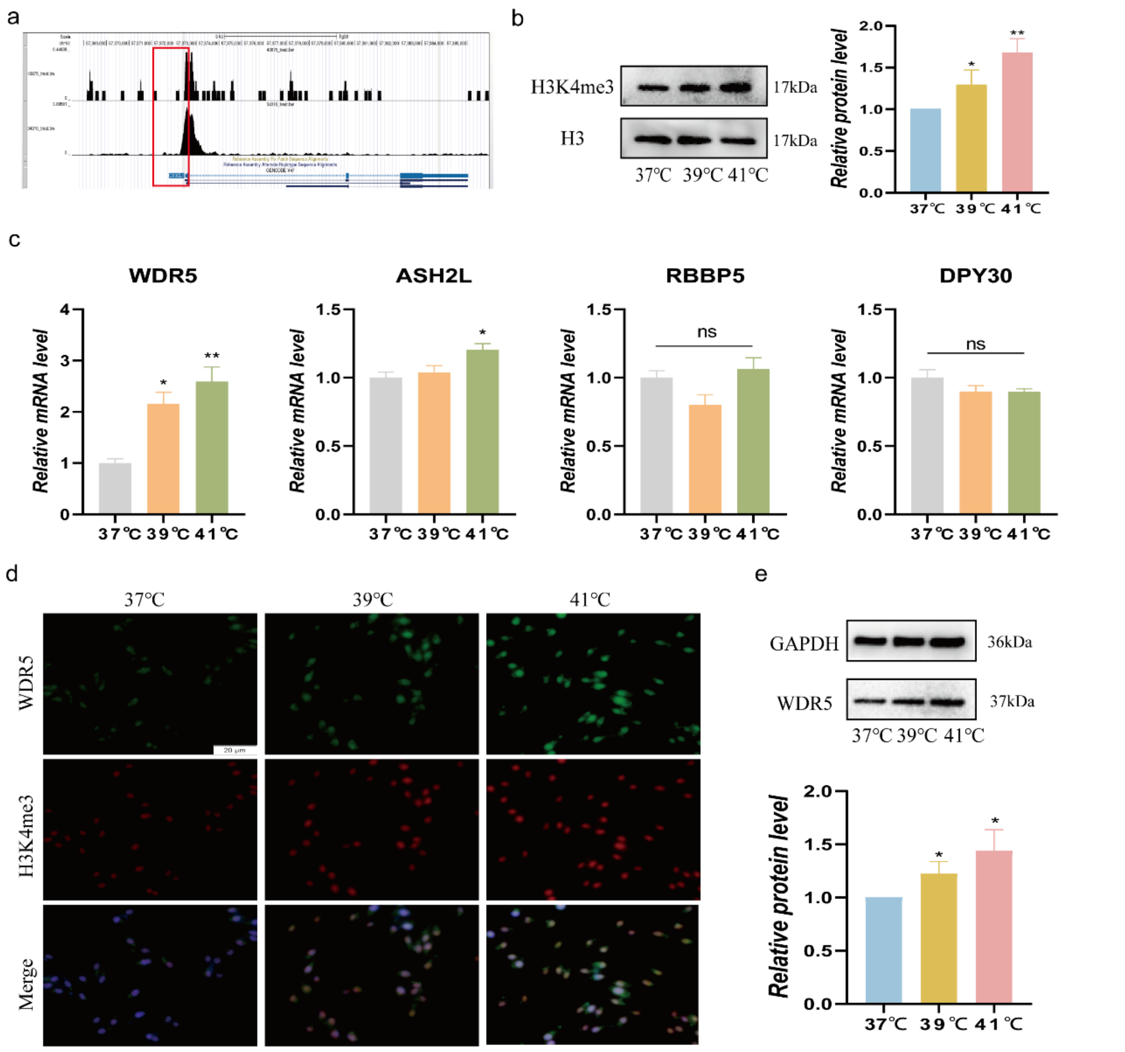

Figure S8. Heat stress upregulates the expression of H3K4me3 and WDR5. (a) The analysis results of the Cistrome DB database. (b-e) After MNT1 cells were exposed to heat stress for 3 days: the expression of H3K4me3 in cells were detected by Western blot (b); The mRNA expression of WDR5, ASH2L, RBBP5 and DPY30 were detected by qRT-PCR(c); IF detects the expression of H3K4me3 and WDR5 in cells and their co-localization(d); The protein expression of WDR5 was detected by Western blot(e). Scale=20μm. Statistical analysis was conducted via a one-way ANOVA with multiple comparisons, mean ± SEM, n = 3 (ns: no significant difference, *P<0.05, **P<0.01).

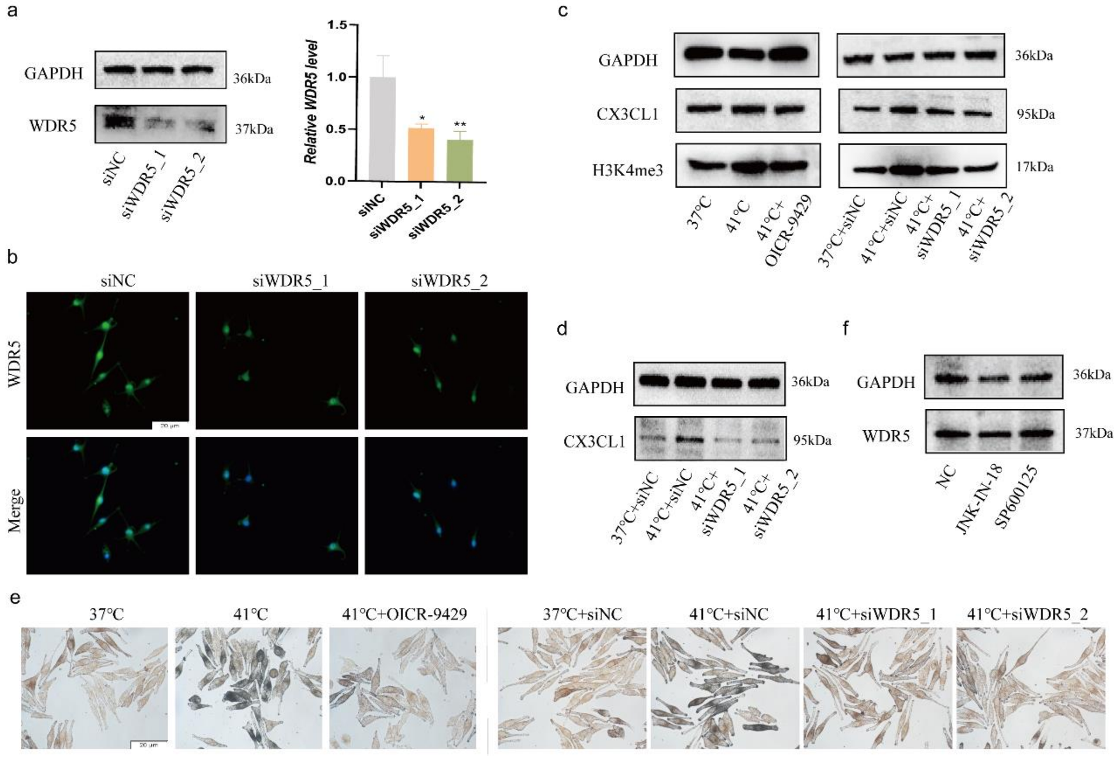

Figure S9. Heat stress modulates the CX3CL1 expression by regulating WDR5-mediated H3K4me3 modification. (a-b) MNT1 and MC cells transfected with si_WDR5. WDR5 expression in MNT1 cells assessed by qRT-PCR and Western blot after 24h and 48h, respectively(a); WDR5 expression in MC cells detected by IF after 48h (b). (c-d) After the MNT1 (c) and MC (d) cells pretreated with OICR-9429 for 30min or transfected with siWDR5 were exposed to heat stress for 3 days, the expression of H3K4me3 or CX3CL1 was detected by Western blot. (e) After the MNT1 cells pretreated with OICR-9429 for 30min or transfected with siWDR5 were exposed to heat stress for 3 days, representative images of MNT1 cells stained with Fontana-Masson showing melanin granules. (f) After MNT1 cells were treated with JNK inhibitors for 48h, the expression of WDR5 in the cells was detected by Western blot. Scale=20μm. Statistical analysis was conducted via a one-way ANOVA with multiple comparisons, mean ± SEM, n = 3 (*P<0.05, **P<0.01).

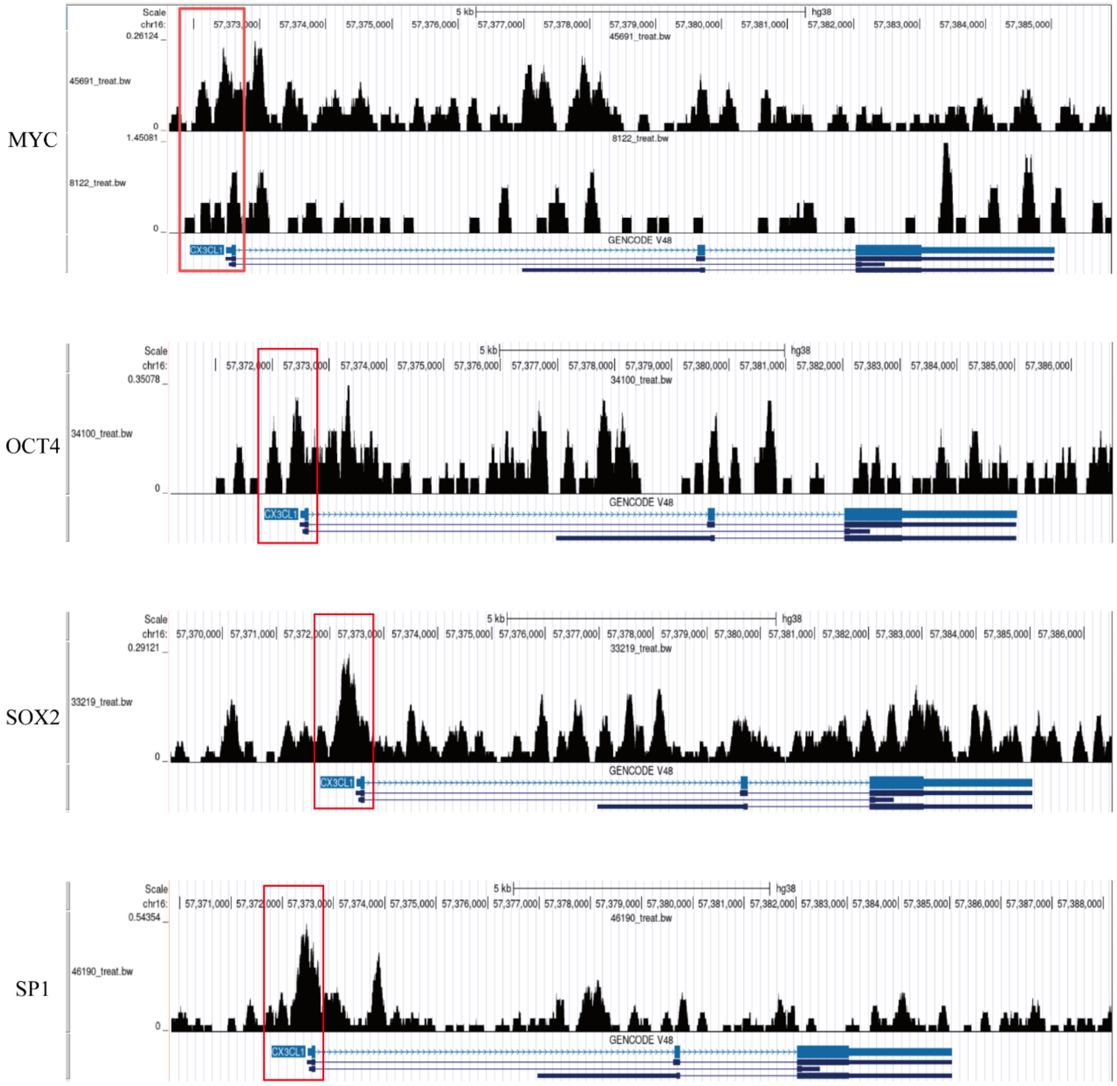

Figure S10. The enrichment level of WDR5-interacting transcription factors in the CX3CL1 promoter region. The enrichment of known transcription factors interacting with WDR5 in the CX3CL1 promoter region was analyzed using the Cistrome DB database.

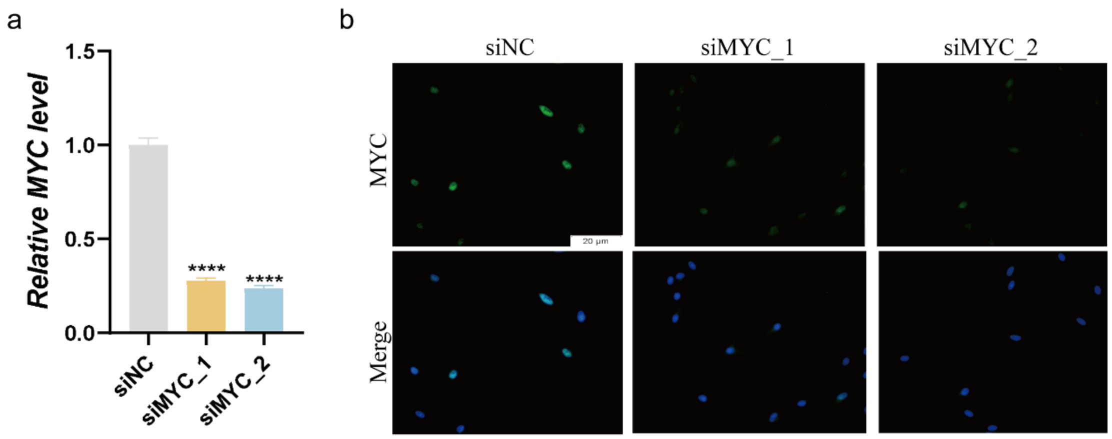

Figure S11. The knockdown efficiency of siMYC in cells. (a-b) MNT1 and MC cells were transfected with si_MYC, MYC expression in MNT1 was detected by qRT-PCR after 24h (a), while its expression in MC was detected by IF after 48h (b). Scale=20μm. Statistical analysis was conducted via a one-way ANOVA with multiple comparisons, mean ± SEM, n = 3 (****P<0.01).
